# Selective binding of choline by a phosphate-coordination-based triple helicate featuring an aromatic box

**DOI:** 10.1038/s41467-017-00915-8

**Published:** 2017-10-16

**Authors:** Chuandong Jia, Wei Zuo, Dong Yang, Yanming Chen, Liping Cao, Radu Custelcean, Jiří Hostaš, Pavel Hobza, Robert Glaser, Yao-Yu Wang, Xiao-Juan Yang, Biao Wu

**Affiliations:** 1 Key Laboratory of Synthetic and Natural Functional Molecule Chemistry of the Ministry of Education, College of Chemistry and Materials Science, Northwest University, 710069 Xi’an, China; 2 Chemical Sciences Division, Oak Ridge National Laboratory, Oak Ridge, TN 37831-6119 USA; 3 Institute of Organic Chemistry and Biochemistry, 16010 Prague 6, Czech Republic; 4 Department of Physical and Macromolecular Chemistry, Faculty of Science, Charles University in Prague, Albertov 6, 12843 Czech Republic; 5 Department of Physical Chemistry, Regional Centre of Advanced Technologies and Materials, Palacký University, 77146 Olomouc, Czech Republic; 6 Department of Chemistry, Ben-Gurion University of the Negev, 84105 Beer-Sheva, Israel

## Abstract

In nature, proteins have evolved sophisticated cavities tailored for capturing target guests selectively among competitors of similar size, shape, and charge. The fundamental principles guiding the molecular recognition, such as self-assembly and complementarity, have inspired the development of biomimetic receptors. In the current work, we report a self-assembled triple anion helicate (host **2**) featuring a cavity resembling that of the choline-binding protein ChoX, as revealed by crystal and density functional theory (DFT)-optimized structures, which binds choline in a unique dual-site-binding mode. This similarity in structure leads to a similarly high selectivity of host **2** for choline over its derivatives, as demonstrated by the NMR and fluorescence competition experiments. Furthermore, host **2** is able to act as a fluorescence displacement sensor for discriminating choline, acetylcholine, l-carnitine, and glycine betaine effectively.

## Abstract (web summary)

The choline-binding protein ChoX exhibits a synergistic dual-site binding mode that allows it to discriminate choline over structural analogues. Here, the authors design a biomimetic triple anion helicate receptor whose selectivity for choline arises from a similar binding mechanism.

## Introduction

Choline is an essential precursor for the synthesis of various important bio-functional molecules, such as the neurotransmitter acetylcholine, membrane lipid phosphatidylcholine, and osmoprotectant glycine betaine^1, 2^. Selective binding of choline is the first step of the biosynthesis of these substances. In *Sinorhizobium meliloti*, a plant root-associated bacterium, the choline-binding protein ChoX has evolved to capture choline among competitors such as acetylcholine and glycine betaine^2^. Given the fact that all these compounds possess the same trimethylammonium head and a slightly different tail, their specific binding is quite challenging. The molecular basis for choline recognition was revealed by the crystal structure of Ch^+ ^⊂ ChoX (Protein Data Bank code 2reg)^2^, where an “aromatic box” (site-I) provides the main binding affinity for the trimethylammonium head through cation–π interactions^3–7^, and two carboxyl groups (site-II) generate the critical differences in affinity for discriminating these analogues. Such a dual-site-binding mode is a common feature of various binding proteins for choline and its derivatives^8–10^.

The pursuit for biomimetic receptors is an important engine driving the progress of supramolecular chemistry^11, 12^. To this end, the naturally assembled protein cavity, which is essential for guest binding, has inspired the synthesis of artificial mimics, either through self-assembly of simple, well-designed building units^13–16^ or through organic synthesis^17–22^. Development of this field is driven by promising applications^23^ of these systems in supramolecular catalysis^24–29^, protein binding^3, 30^, cellar imaging^31^, and bio-process monitoring^32^. Although strong binding of choline by artificial receptors has been widely reported^33–35^, good selectivity for choline has not yet been achieved^36–38^. In nature, the ChoX protein is a specific high-affinity choline transporter and various choline derivatives present no or much weaker binding affinity. For instance, its binding for choline is 54-fold stronger than for acetylcholine, which is the strongest competitor^1a^. However, most artificial mimics display no obvious discrimination between them, and the reported selectivity (*K*_choline_/*K*_acetylcholine_), to the best of our knowledge, is no more than three^37^.

We have recently developed a new strategy towards the construction of supramolecular architectures, wherein anion coordination chemistry^39–42^ is employed instead of the widely utilized metal coordination to drive the supramolecular assembly^43–49^. Various supramolecular structures have been obtained, including triple helicates^43, 44^ and tetrahedral cages^45, 46^. The negatively charged cavity make these anion-based assemblies potential receptors for positively charged species, including important biomolecules such as choline and other methylated substrates^3, 50^.

Herein, we report a phosphate coordination-based triple helicate receptor functionalized with an aromatic box resembling the dual-site-binding mode of ChoX protein to achieve enhanced selectivity to choline. This receptor is developed as a fluorescence displacement sensor capable of discriminating choline from other structural analogues, which paves a way for potential applications in biological systems.

## Results

### Crystal structure of host 1

**Fig. 1:**
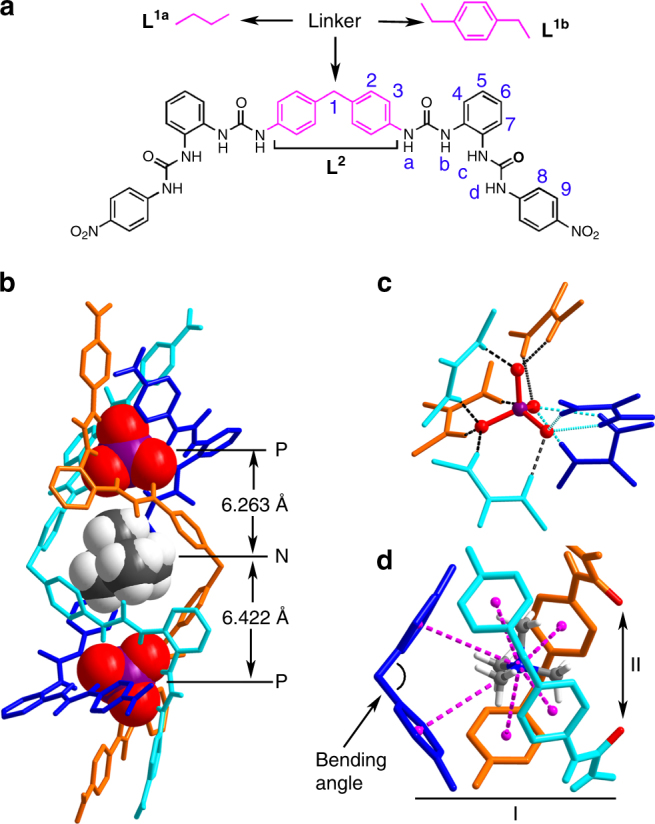
Structure of a ChoX-mimicking triple anion helicate receptor. **a** Structures of bis-bisurea ligands **L** ^**1a**^, **L** ^**1b**^, and **L** ^**2**^ that can readily form the A_2_L_3_ (A = anion, L = ligand) triple helicates when coordinated with PO_4_^3−^ ions. **b** Crystal structure of complex **1**, (TMA)_5_[(TMA) ⊂ (PO_4_)_2_(**L** ^**2**^)_3_] (⊂ = encapsulated by; only an *M* enantiomer is shown; other counter cations, solvent molecules, and non-acidic protons are omitted for clarity); **c** Hydrogen bonds formed between a PO_4_^3−^ ion and six urea units; **d** The aromatic box (site-I) trapping a TMA^+^ through cation–π interactions (purple-dashed lines) and a potential hydrogen-bonding site (II), which together resembles the structure of Ch^+ ^⊂ ChoX

Our previous studies^43, 44^ demonstrated that the properly spaced bis-bisurea ligands **L** ^**1a**^ and **L** ^**1b**^ (Fig. 1a) can readily form the A_2_L_3_ (A = anion, L = ligand) triple helicates when coordinated with PO_4_^3−^ ions. As a continuous effort to functionalize this type of assembly, ligand **L** ^**2**^ was synthesized to create an aromatic box with its “V” shaped 4,4′-methylenebis(phenyl) linker upon formation of the helicate. This approach proved successful, leading to the crystal structure of complex **1**, (TMA)_5_[(TMA) ⊂ (PO_4_)_2_(**L** ^2^)_3_] (Fig. 1b), comprising a triple helicate that contains a binding cavity flanked by six phenyl rings of the three 4,4′-methylenebis(phenyl) linkers, which traps a tetramethylammonium (TMA^+^) cation inside. Each PO_4_^3−^ center is coordinated by six urea units (Fig. 1c), four of which (shown in turquoise and orange) are arranged optimally along edges of the tetrahedral anion, and the remaining two (shown in blue) alternatively occupy two adjacent oxygen vertices, forming overall 12 hydrogen bonds to phosphate (dashed lines, N∙∙∙O distances range from 2.681 to 3.213 Å, average 2.796 Å; N−H∙∙∙O angles from 141° to 178°, average 156°; see Supplementary Table 1). The encapsulated TMA^+^ ion is positioned close to the middle point of the two PO_4_^3−^ ions (with P∙∙∙N distances of 6.263 and 6.422 Å, Fig. 1b). Besides the electrostatic interactions, TMA^+^ guest is further stabilized by cation–π interactions with the six phenyl rings of the linkers (purple-dashed lines, N∙∙∙centroid distances: 4.295−4.808 Å, average 4.535 Å, Fig. 1d, see also Supplementary Fig. 1). Noticeably, the triple helical structure of the inclusion complex **1** is not C_3_-symmetric and its cavity appears as a bowl-shaped pocket, where one of the ligands (Fig. 1d, blue, bending angle 114°) defines the bottom and the other two (turquoise and orange, bending angle 112°) circle a mouth decorated by two urea carbonyl groups (5.091 Å apart).

**Fig. 2:**
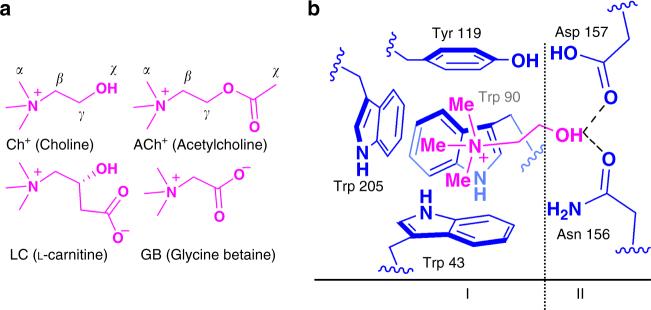
The recognition mode of choline-binding protein ChoX. **a** Structures of choline and its derivatives, showing that they possess the same trimethylammonium head and a slightly different tail. **b** ChoX exhibits a synergistic dual-site-binding mode, where an “aromatic box” (site-I) provides the binding affinity for the trimethylammonium head, and two carboxyl groups (site-II) generate the critical differences in affinity for discriminating structural analogues^2^

It was noticed that while TMA^+^ is same with the trimethylammonium head of choline and its derivatives (Fig. 2a), the structure of complex **1** resembles that of Ch^+ ^⊂ ChoX (Fig. 2b) with an aromatic box (I, six phenyl groups) and a hydrogen-bonding site (II) and therefore prompted us to investigate its binding properties towards choline and its derivatives.

### Host–guest studies of host 2

Considering that the counter cation TMA^+^ in complex **1** will introduce competition, a new complex **2** [TBA]_6_[(PO_4_)_2_(**L** ^**2**^)_3_] (host **2**) was thus prepared, wherein TBA^+^ (tetrabutylammonium) was employed as the counter cation, which is too large to be encapsulated in the helicate’s cavity (Supplementary Table 2). High-resolution electrospray ionization mass spectrometry (HRMS) confirmed the formation of a complex with the (PO_4_)_2_(**L** ^**2**^)_3_ stoichiometry. Specifically, the observed *m*/*z* peak for [(**L** ^2^)_3_(PO_4_)_2_(TBA)_2_H_2_]^2−^ (1530.1376) is nearly identical to the predicted value (1530.1322), whereas its isotopic-splitting pattern is also in excellent agreement with that from the simulation (Supplementary Fig. 2).

**Fig. 3:**
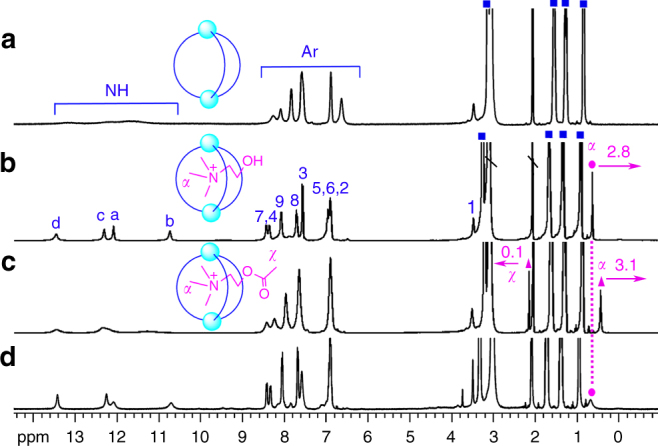
^1^H NMR spectra demonstrating complexation of different guests by host **2**. **a** ^1^H NMR (acetone-*d*_6_/1.5% H_2_O, 400 MHz) spectra of host **2** alone and in the presence of 1 equiv. of **b** Ch•Cl, **c** ACh•Cl, and in coexistence of 1 equiv. of **d** Ch•Cl, ACh•Cl, LC, and GB (blue squares TBA^+^, magenta circles Ch^+^, magenta triangles ACh^+^, \ solvent residue; insets show representative pictures of free host **2** and host–guest inclusion complexes)

The binding of choline and potential competitors by host **2** was evaluated by ^1^H NMR spectroscopy in acetone-*d*_6_/1.5% H_2_O, by adding 1 equiv. of choline (Ch^+^), acetylcholine (ACh^+^), glycine betaine (GB) and l-carnitine (LC), respectively (Fig. 3). Host **2** alone showed a set of broad signals (Fig. 3a), whereas in the presence of one equivalent of Ch^+^, all signals turned sharp and that of trimethylammonium protons (Hα) experienced a significant upfield shift of 2.8 ppm, indicating that Ch^+^ was encapsulated in the shielding aromatic cavity of host **2** (Fig. 3b). Two-dimensional NMR spectroscopy provided further evidence for the encapsulation by the clear ^1^H-^1^H NOE (nuclear Overhauser effect) correlations between Hα of Ch^+^ and the phenylene protons (H3) of the linker of **L** ^**2**^ (see Fig. 1a for proton numbering, Supplementary Figs. 3 and 4). Moreover, HRMS confirmed the identity of this host–guest assembly by a group of *m*/*z* peaks of [(**L** ^2^)_3_(PO_4_)_2_(TBA)_*x*_(Ch)_*y*_H_*z*_]^2−^ (*x*, *y*, *z* = 0, 1, 2, or 3) (Supplementary Fig. 5).

Among the other three tested guests, GB and LC showed no significant binding (Supplementary Fig. 6) and only ACh^+^ induced obvious changes in the NMR spectrum though the resulted signals remained broad (Fig. 3c), which is indicative of low symmetry of the host–guest complex. The proton signal of the trimethylammonium fragment (Hα) of ACh^+^ shifted upfield by 3.1 ppm, whereas the methyl protons of the acetyl group (Hχ) showed a slight downfield shift (Δ*δ* = 0.1 ppm), implying that this “tail” group is positioned out of the shielding cage. In both cases of Ch^+^ and ACh^+^, the formation of a single host–guest species was confirmed by DOSY (diffusion-ordered nuclear magnetic resonance spectroscopy) spectra, in which the diffusion coefficients of the guest and host **2** were very similar (Supplementary Figs. 7, 8). In addition, competition experiments showed that the spectrum of host **2**/Ch^+^ was only slightly altered by the addition of one equiv. of competitors ACh^+^, GB, and LC, indicating that Ch^+^ is the most favorable guest of host **2** among these four biomolecules (Fig. 3d).

**Fig. 4:**
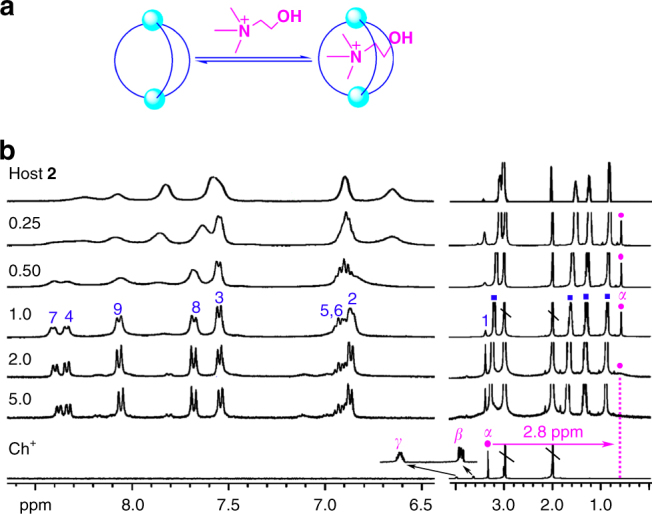
Studies of the complexation process of choline by host **2**. **a** Scheme of the equilibrium involved in the binding of Ch•Cl by host **2**. **b** Stacking ^1^H NMR (acetone-*d*_6_/1.5% D_2_O, 400 MHz) spectra of host **2** (TBA)_6_[(PO_4_)_2_(**L**)_3_] alone and in the presence of Ch•Cl (equiv. is labeled by numbers), and the spectrum of free Ch•Cl (blue squares TBA^+^, magenta circles Ch^+^, \ solvent residue)

Titration experiments revealed that either Ch^+^ (Fig. 4) or ACh^+^ (Supplementary Fig. 9) was bound in a 1:1 (host:guest) binding mode with a similar intermediate exchange on the NMR timescale^51^. Taking Ch^+^ as an example, in the first step within the addition of 0-1 equiv. of Ch^+^, increasing amounts of the free host **2** bound the Ch^+^ as demonstrated by gradual shifting of its broad signals that became sharp and saturated when guest/host ratio reached 1:1. Meanwhile, the methyl proton signal of Ch^+^ kept sharp and stayed at 0.6 ppm, indicating this guest ion is predominantly encapsulated in the cage of **2**. In the second step when gradually increasing the amount of Ch^+^ from 1 to 5 equiv., the signals of host **2** showed no significant changes, whereas the Hα signal of Ch^+^ at 0.6 ppm turned broad until it disappeared, implying that the excessive Ch^+^ guest ions remained outside of the cage and engaged in intermediate exchange with the encapsulated ones.

### Binding constants determination

Such intermediate exchange properties in the binding of Ch^+^ and ACh^+^ prohibit the direct determination of association constants by fitting a titration curve (in the case of fast exchange) or by estimating the concentrations of host and guest based on integral ratios (slow exchange). Hence, to quantitatively evaluate the selectivity of host **2** for Ch^+^ over ACh^+^, it is necessary to carry out competition experiments with a suitable guest as the reference, which should have a determinable association constant and is bound by host **2** with comparable strength as Ch^+^ and ACh^+^. With this in mind, three guest ions TMA^+^, TEA^+^, and TPA^+^ were screened by titration with host **2**. TMA^+^ was found to bind in intermediate exchange (Supplementary Figs. 10–12) and TPA^+^ showed no obvious binding (Supplementary Fig. 13). Fortunately, TEA^+^ was bound in fast exchange (Supplementary Figs. 14–16), and the association constant was determined as *K* > 10^4^ M^−1^ (the limit for accurate determination using this method) by fitting the shift profile of the CH_3_ proton to a 1:1 mode with WinEQNMR program (Supplementary Figs. 17, 18)^52^. By taking TEA^+^ as a standard guest, the binding constants of TMA^+^, Ch^+^, and ACh^+^ relative to TEA^+^ were estimated to be 17.4 *K*(TEA^+^), 24.1 *K*(TEA^+^), and 1.2 *K*(TEA^+^), respectively (Supplementary Table 3; Supplementary Fig. 19). Thus the binding affinity of Ch^+^ by host **2** appears to be about 20-fold stronger than that of ACh^+^. On the other hand, the binding of GB and LC was too weak to compete with TEA^+^ (*K *« *K*(TEA^+^)), and the selectivity of host **2** for the four was in the order of Ch^+^ > ACh^+^ >> GB, LC.

**Fig. 5:**
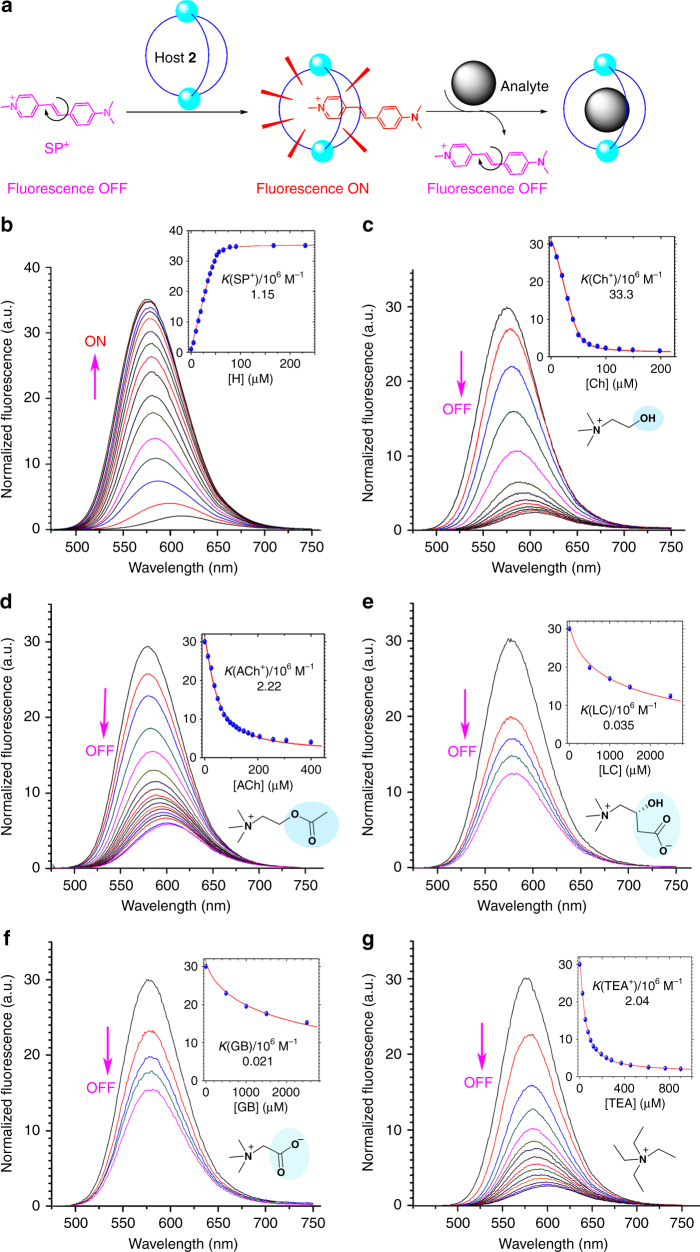
Determination of binding constants of host **2** for guests by fluorescence titrations. **a** Principle of fluorescence “switch-off” displacement assay for analyte sensing. **b** Emission spectra of 50 μM SP•I before and after addition of aliquot equiv. of host **2** (**H2**), showing a fluorescence “switch on” response. **c** Emission spectra of SP^+^/**H2** (50 μM/50 μM) before and after addition of aliquot equiv. of Ch•Cl, **d** ACh•Cl, **e** LC, **f** GB, and **g** TEA•Cl, respectively, showing a fluorescence “switch-off” response. All spectra were recorded in acetone/1.5% H_2_O with *λ*_ex_ = 470 nm. Insets show structures of analytes with the “tail” highlighted, and the corresponding association constants determined by fitting the titration curves at *λ*_em_ = 575 nm (blue dots) to a 1:1 (host : guest) binding mode by the Dynafit program (error < 10%)

**Fig. 6:**
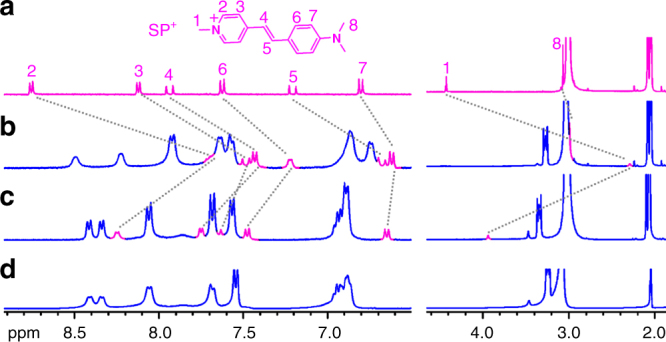
Displacement binding of SP^+^ by Ch^+^. **a** ^1^H NMR (400 MHz, acetone-*d*_6_/1.5% H_2_O) spectra of **a** 1 mM SP•I (inset shows the structure), **b** host **2**/SP•I (1/1 equiv.), **c** host **2**/SP•I/Ch•Cl (1/1/1 equiv.), and **d** host **2**/Ch•Cl (1/1 equiv.). Chemical shifts of protons of SP^+^ upon complexation and decomplexation were labeled by gray dashed lines

Moreover, efforts were also devoted to calculate the association constants of host **2** with the guests in a more accurate way based on fluorescence titrations. As neither host **2** nor the tested guests are fluorescent, a fluorescence displacement sensor was developed based on the inclusion complex, SP^+ ^⊂ **2**, (SP^+^ = 4-(4′-dimethylamino)styryl-1-methylpyridinium), which can signal guest binding by displacement of the SP^+^ (Fig. 5a)^53^. The formation of host **2**–SP^+^ complex and subsequently displacement of SP^+^ by Ch^+^ were firstly confirmed by ^1^H NMR spectra (Fig. 6; Supplementary Table 4). The pyridinium N-methyl signal (H1) of SP^+^ experienced a significant upfield shift of 2.1 ppm, indicating SP^+^ was encapsulated in the shielding aromatic cavity of host **2**. Phenyl and alkenyl signals of SP^+^ also experienced dramatic upfield shifts (H2–7) 0.2–1.1 ppm), whereas the proton signals of dimethylamino (H8) showed only a slight upfield shift (0.08 ppm), suggesting that this “tail” group is positioned out of the shielding cage. The encapsulated SP^+^ was readily replaced by one equiv. of subsequently added Ch^+^, which induced mainly recovery of the signals of the free SP^+^ and formation of a Ch^+ ^⊂ host **2** complex.

Fluorescence titration of a 50 μM solution of SP^+^ with host **2** was then performed, which induced an increase of the fluorescence by 35-fold (fluorescence ON) and a 37 nm hypsochromic shift (612 nm to 575 nm) of the maximum emission (Fig. 5b). The *K*(SP^+^) was calculated as 1.15 × 10^6 ^M^−1^ by fitting the titration curve of SP^+ ^⊂ **2** with Dynafit program^54^ to a 1:1 mode, which was further confirmed by the Job’s plot (Supplementary Fig. 20). Upon addition of tested guests to a solution of SP^+^/host **2** (50 μM/50 μM), the fluorescence decreased (OFF) as a result of displacement of SP^+^ and the corresponding association constants were thus obtained by fitting the titration curves to the 1:1 mode. The *K*(Ch^+^) and *K*(ACh^+^) (Fig. 5c, d) were 3.33 × 10^7^ M^−1^ and 2.22 × 10^6^ M^−1^, respectively, giving a selectivity of *K*(Ch^+^)/*K*(ACh^+^) as 15, which is consistent with the value (20) obtained from NMR. Moreover, the high sensitivity of fluorescence changes (Fig. 5e, f) enables determination of *K*(GB) and *K*(LC), which as mentioned above could not be measured by NMR competition with TEA^+^, as 2.1 × 10^4 ^M^−1^ and 3.5 × 10^4 ^M^−1^, respectively, and the selectivity was ranked as Ch^+^ > ACh^+^ >> GB ≈ LC. In addition, the *K*(TEA^+^) and *K*(TMA^+^) were determined as 2.04 × 10^6 ^M^−1^ and 1.74 × 10^7 ^M^−1^, respectively (Fig. 5g; Supplementary Fig. 21). These results match well with those obtained from NMR competition titrations (Supplementary Table 5).

### Mechanism studies

**Fig. 7:**
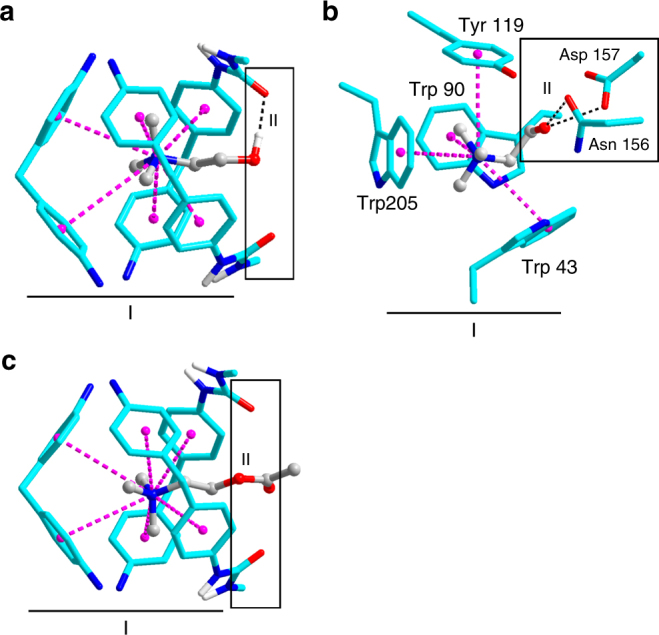
Comparison of the binding modes for Ch^+^ and Ach^+^ in host **2** and ChoX. **a** DFT-optimized structure of Ch^+ ^⊂ **2**. **b** Crystal structure of Ch^+ ^⊂ ChoX (Protein Data Bank code 2reg)^2^. **c** DFT-optimized structure of ACh^+ ^⊂ **2**. Binding sites are labeled as I and II, cation–π, and hydrogen bonding interactions are labeled as purple-dashed line and black-dashed line, respectively

The origin of the high selectivity of host **2** towards Ch^+^ over ACh^+^ was further investigated by DFT optimization of the structures of the corresponding inclusion complexes. Ch^+ ^⊂ **2** (Fig. 7a) demonstrates a similar dual-site-binding mode to that of Ch^+ ^⊂ ChoX (Fig. 7b)^2^, whereas ACh^+ ^⊂ **2** presents only a single binding at site-I but not at site-II, which was accounted for the discrimination of Ch^+^ and ACh^+^ (Fig. 7c) Specifically, at site-I of Ch^+ ^⊂ **2**, the aromatic box is composed of six phenyl groups with N∙∙∙centroid distances ranging from 4.3 to 4.9 Å (av. 4.7 Å, Supplementary Fig. 22), and in Ch^+ ^⊂ ChoX, the cage is surrounded by the aryl rings of three tryptophans and a single tyrosine residue (Trp43, Trp90, Trp205, and Tyr119) with N∙∙∙centroid distances of 4.2 to 4.5 Å (av. 4.3 Å, Supplementary Fig. 23). At site-II of Ch^+ ^⊂ **2**, the hydrogen bond shows an O∙∙∙O distance of 2.7 Å and O−H∙∙∙O angle of 168°. Correspondingly in Ch^+ ^⊂ ChoX, there are two hydrogen bonds with O∙∙∙O distances of 2.7 and 3 Å, respectively (O−H∙∙∙O angles not available). The DFT-optimized structure of the ACh^+ ^⊂ **2** displays a similar aromatic box encapsulating the trimethylammonium head and the methyl protons of the acetyl group are extending out of the cavity, which is consistent with the results demonstrated by ^1^H NMR (Fig. 3c). The N∙∙∙centroid distances in ACh^+ ^⊂ **2** are ranging from 4.3 to 4.9 Å with an average of 4.6 Å (Supplementary Fig. 24). In contrast to Ch^+ ^⊂ **2**, no hydrogen bond is formed in ACh^+ ^⊂ **2** with the tail group, which results in a 2.8 kcal mol^−1^ weaker binding than that of Ch^+^ (Supplementary Table 6). On the other hand, it is reasonable to hypothesize that binding of the zwitterionic guests, LC and GB, in a similar mode to that of Ch^+^ should position the guest’s carboxylate group in proximity to the negatively charged cavity and the two urea carbonyl residues (site-II). The resulting strong electrostatic repulsion is possibly responsible for the much weaker binding of GB and LC.

It should be noted that though DFT modeling has provided evidences that the selectivity of host **2** for Ch^+^ over ACh^+^ may arise from the additional secondary binding with the hydroxyl “tail”, another factor, i.e., the smaller steric effect of Ch^+^ than ACh^+^ cannot be ruled out. To address this point, the TMA^+^ was selected as a model for control experiments, which possesses a trimethylammonium head same as Ch^+^, whereas a smaller tail (–Me vs –CH_2_CH_2_OH). If there is no hydrogen bonding at site-II, the binding of TMA^+^ is supposed to be stronger than Ch^+^ due to its smaller steric effect. On the contrary, the association constant was determined as *K*(TMA^+^) = 0.7 *K*(Ch^+^) by NMR and fluorescence titrations (Supplementary Fig. 21; Supplementary Table 5). This was rationalized by the proposed secondary hydrogen bonding at site-II, which provides positive binding energy to overcome the negative steric effect and induces a stronger binding than TMA^+^.

## Discussion

In conclusion, we report a phosphate coordination-based triple helicate as a type of supramolecular host with an aromatic box for guest binding. Of particular significance is this host system displays a unique high selectivity towards choline over the closely related acetylcholine (*K*_choline_/*K*_acetylcholine_ > 15). Mechanism studies demonstrated that the synergetic dual-site binding of both the trimethylammonium head and hydroxyl-tail is the most critical reason for its selectivity. On the basis of this property, the triple anion helicate has been developed as a fluorescence displacement sensor capable of effectively discriminating choline, acetylcholine, l-carnitine and glycine betaine. We are currently modifying the system to exploit its potential applications in cellar imaging^31^, monitoring biomembrane transport^32^, recognition of proteins^55^, and tracking enzyme’s activity^56–59^, etc.

## Methods

### General information

All reagents were obtained commercially and used without further purification. The indicator dye, 4-(4-dimethylaminostyryl)-1-methylpyridinium iodide (SP•I), was synthesized following a published procedure^60^. All NMR spectra were obtained at 20 °C by using Bruker AVANCE III-400 MHz or −500 MHz spectrometers. ^1^H and ^13^C NMR chemical shifts were reported relative to residual solvent peaks (^1^H NMR: 2.50 ppm for DMSO-*d*_6_, 2.05 ppm for acetone-*d*_6_; ^13^C NMR: 39.52 for DMSO-*d*_6_). Infrared (IR) spectra were recorded on a Bruker EQUIOX-55 spectrometer. High-resolution mass spectra were performed with an Orbitrap mass spectrometer (Q-Exactive, Thermo Scientific, San Jose, CA) for complex **1** and a Bruker micrOTOF-Q II ESI-Q-TOF LC/MS/MS spectrometer for complex **2** and complex **2**/1 equiv. Ch•Cl. Fluorescence spectra (λ_ex_ = 470 nm) were recorded on a Horiba FL1039A/40 A spectrophotometer in a 1 cm quartz cell.

### Synthesis of **L**^**2**^

1-(2-Aminophenyl)-3-(4-nitrophenyl)urea^44^ (1.07 g, 3.9 mmol) and methylene di-*p*-phenylene diisocyanate (0.47 g, 1.9 mmol) in 200 ml THF were refluxed overnight and the precipitate was filtered off and washed several times with acetone and diethyl ether and then dried over vacuum to get the analytically pure product as a yellow powder (1.33 g, 88%). ^1^H NMR (400 MHz, DMSO-*d*_*6*_, ppm): *δ* 9.84 (s, 1H, NH), 9.00 (s, 1H, NH), 8.26 (s, 1H, NH), 8.19 (d, *J* = 8.0 Hz, 2 H, H9), 8.06 (s, 1H, Hd), 7.71 (d, *J* = 8.0 Hz, 2H, H8), 7.64 (d, *J* = 8.0 Hz, 1H, H7), 7.54 (d, *J* = 8.0 Hz, 1H, H4), 7.37 (d, *J* = 8.0 Hz, 2H, H3), 7.11 (m, 4H, H2, H5, H6), 3.80 (s, 1H, H1). ^13^C NMR (100 MHz, DMSO-*d*_6_): 153.21 (CO), 152.78 (CO), 146.62 (C), 140.98 (C), 137.67 (C), 135.12 (C), 132.20 (C), 130.17 (C), 128.96 (CH), 125.19 (CH), 124.88 (CH), 123.83 (CH), 123.60 (CH), 118.50 (CH), 117.44 (CH) (Supplementary Fig. 25). IR (KBr, ν/cm^−1^): 3266 (NH), 1653 (CO), 1588, 1558, 1498, 1398, 1313, 1299 (NO_2_), 1234, 1179, 1099, 839, 749, 680, 625. HRMS: *m*/*z* Calcd. for [M–H], 793.2483, found 793.2471.

### Self-assembly of (TMA)_5_[(TMA) ⊂ (PO_4_)_2_(L^2^)_3_] (complex **1**)

**L** ^**2**^ (108 mg, 0.136 mmol) was reacted with 0.67 equiv. of (TMA)_3_PO_4_ (181 µl 0.5 M solution, generated in situ from (TMA)OH and H_3_PO_4_) in acetonitrile (5 ml). After stirring overnight at room temperature, a clear yellow solution was obtained. Slow vapor diffusion of diethyl ether into this solution provided yellow crystals of the inclusion complex **1** within one week (126 mg, 92%). The identity of complex **1** was confirmed by HRMS (Supplementary Fig. 26).

### Self-assembly of (TBA)_6_[(PO_4_)_2_(L^2^)_3_] (complex **2**)

**L** ^**2**^ (122 mg, 0.154 mmol) was reacted with 0.67 equiv. of (TBA)_3_PO_4_ (205 µl 0.5 M solution, generated in situ from (TBA)OH and H_3_PO_4_) in acetonitrile (5 ml). After stirring overnight at room temperature, a clear yellow solution was obtained. Pouring this solution into 50 ml of diethyl ether induced a yellow precipitate, which was collected through filtration and then dried over vacuum to get complex **2** as a yellow powder (182 mg, 88%). The identity of complex **2** was confirmed by HRMS (Supplementary Fig. 2).

### Crystallography and data analysis

Diffraction data were collected on a Bruker SMART APEX II diffractometer at 100 K with graphite-monochromated Mo Kα radiation (*λ* = 0.71073 Å). An empirical absorption correction using SADABS was applied for the data. The structures were solved by direct methods using the SHELXS-2014 program. All non-hydrogen atoms were refined anisotropically by full-matrix least-squares on *F* ^2^ by the use of the program SHELXL-2014, and hydrogen atoms were included in idealized positions with thermal parameters equiv. to 1.2 times those of the atom to which they were attached.

### NMR titrations

Given the poor solubility of the cationic guests (all used as chloride salts unless otherwise noted) in acetone, a stock solution of host complex **2** (10 mM, 100 mM in acetone-*d*_6_/1.5% D_2_O) was titrated to a 2 mM solution of the guest (TMA^+^, TEA^+^, Ch^+^, Ach^+^) in acetone-*d*_6_/1.5% D_2_O. Equivalents of the host were titrated as 0.2, 0.5, 1, 2, and 4, and thus the titration spectra were obtained with equiv. of the guest as 5, 2, 1, 0.5, and 0.25. Other samples for 2D NMR experiments (NOESY, DOSY, and correlation spectroscopy = COSY) were prepared in acetone-*d*_6_/1.5% H_2_O with a 6 mM solution of complex **2**.

### Fluorescence titrations

Fluorescence titrations were performed at room temperature. In the fluorescence titrations of SP•I (dye) with host **2** (Fig. 5b), known amounts of host **2** were added to a 2 ml solution of 50 μM SP•I in acetone–1.5% H_2_O. To keep the concentration of dye as constant in the course of the titration, 1 mM stock solutions of host were prepared with a 50 μM solution of [SP•I] in acetone–1.5% H_2_O. Similarly in the processes of displacement titrations, known amounts of competitors (analytes) were added successively to a 2 ml solution of [host **2**]/[SP•I] (50 μM/50 μM) in acetone–1.5% H_2_O. To keep the concentration of host **2** and dye constant in the course of the titration, stock solutions of analytes ([TMA•Cl] = 1 mM, [Ch•Cl] = 2 mM, [TEA•Cl] = [ACh•Cl] = 5 mM) were prepared with a solution of [host **2**]/[SP•I] (50 μM/50 μM) in acetone–1.5% H_2_O. As GB and LC require much excessive amounts to induce significant fluorescence changes, the high-concentration stock solutions could not be prepared in [host **2**]/[SP•I] (50 μM/50 μM) in acetone–1.5% H_2_O due to the low solubility. Alternatively, 1.0 M stock solutions in deionized water were utilized, which totally introduced 4 μl H_2_O (0.2% of the 2 ml mother solution) during each titration course and the influence of this tiny amount of water was ignored.

### Binding constants determination

In the NMR titrations, the binding constants of guests, *K*(G), relative to *K*(TEA^+^) were estimated based on competition experiments. In the fluorescence titrations, they were determined by fitting titration curves at *λ*_em_ = 575 nm to a 1:1 (host:guest) binding mode by Dynafit program (error < 10%)^54^.

### Molecular modeling

The DFT method, augmented with empirical dispersion term (D3), was utilized for the current study^61–63^.

### Data availability

The X-ray crystallographic data for the structure of complex **1**, (TMA)_5_[(TMA) ⊂ (PO_4_)_2_(**L** ^2^)_3_], is available at the Cambridge Crystallographic Data Centre (CCDC) with the deposition number CCDC 1517699; Supplementary methods of binding constants determination, molecular modeling, and additional data supporting the findings of this study are available in Supplementary Information file. Cartesian coordinates of the optimized geometries of Ch^+ ^⊂ **2** and ACh^+ ^⊂ **2** are available in Supplementary Data 1.

## Electronic supplementary material

Supplementary Information

Description of Additional Supplementary Information

Supplementary Data 1
