# Supplementary material for: Selective binding of choline by a phosphate-coordination-based triple helicate featuring an aromatic box
Source: Nat Commun. 2017 Oct 16;8:938. doi: 10.1038/s41467-017-00915-8 (PMC5643546; doi:10.1038/s41467-017-00915-8)
Supplement: Supplementary file 1 — Supplementary Information [file 41467_2017_915_MOESM1_ESM.pdf]

Supplementary Table 1. Hydrogen bonds [Å and °] in the crystal structure of complex **1**, $(TMA)_5[(TMA)\subset(PO_4)_2(\mathbf{L}^2)_3]$ ($\subset$ means encapsulated by).

D–H···*A*	*d*(*D*–H)	*d*(H···*A*)	*d*(*D*···*A*)	∠(*D*H*A*)
N2–H2A···O27	0.86	1.92	2.684(6)	148
N3–H3A···O28	0.86	2.12	2.939(7)	159
N4–H4···O28	0.86	1.88	2.710(6)	161
N5–H5A···O25	0.86	1.95	2.798(6)	169
N6–H6A···O31	0.86	1.95	2.785(6)	162
N7–H7···O29	0.86	2.08	2.877(6)	154
N8–H8···O29	0.86	1.93	2.772(6)	164
N9–H9A···O30	0.86	1.87	2.711(6)	166
N12–H12S···O27	0.86	1.86	2.681(6)	160
N13–H154···O26	0.86	2.04	2.831(6)	153
N14–H200···O26	0.86	1.93	2.762(6)	161
N15–H15···O28	0.86	2.00	2.744(6)	145
N16–H16A···O32	0.86	1.94	2.758(5)	158
N17–H17A···O31	0.86	1.97	2.763(6)	152
N18–H18···O31	0.86	1.93	2.756(6)	162
N19–H19A···O30	0.86	1.95	2.759(6)	157
N22–H22···O25	0.86	1.93	2.745(7)	157
N23–H23A···O25	0.86	1.99	2.788(7)	153
N24–H24A···O26	0.86	2.50	3.212(7)	141
N25–H25···O26	0.86	1.84	2.697(6)	178
N26–H26A···O29	0.86	2.00	2.777(5)	150
N27–H27A···O29	0.86	2.21	2.978(6)	149
N28–H28···O32	0.86	2.14	2.869(6)	142
N29–H29···O32	0.86	1.93	2.698(5)	148
Average	0.86	1.99	2.796	156

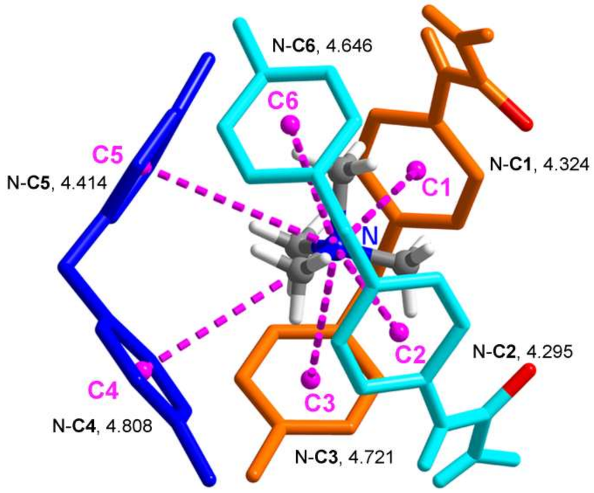

Supplementary Figure 1. Binding sites in crystal structure of $TMA^{+}\subset$**1** with data of N···centroid distances for evaluating the cation-π interactions (purple dashed lines [Å], Average, 4.535 Å).

Supplementary Table 2. Size of quaternary ammoniums.

Cations	TMA^{+}	TEA^{+}	TPA^{+}	TBA^{+}
CSD Hits	760	1410	292	2899
$d_{(N\text{-}CH3)}$	1.484	2.549	3.873	**4.890**
$d_{(N\text{-centroid})}$ in complex **1** ranges from **4.295**–**4.808**, and **4.535** in average.				
Abbreviations: TMA^{+}, tetramethylammonium; TEA^{+}, tetraethylammonium; TPA^{+}, tetrapropylammonium; TBA^{+} = tetrabutylammonium. The Cambridge Structural Database (CSD) searching results of the average N-CH_3 distances [Å] of quaternary ammoniums, demonstrating that the size of TBA^{+} is too big to fit the cage of the triple helicate. The CSD searches were filtered by "R < 0.1, Not disordered, and Not polymeric".				

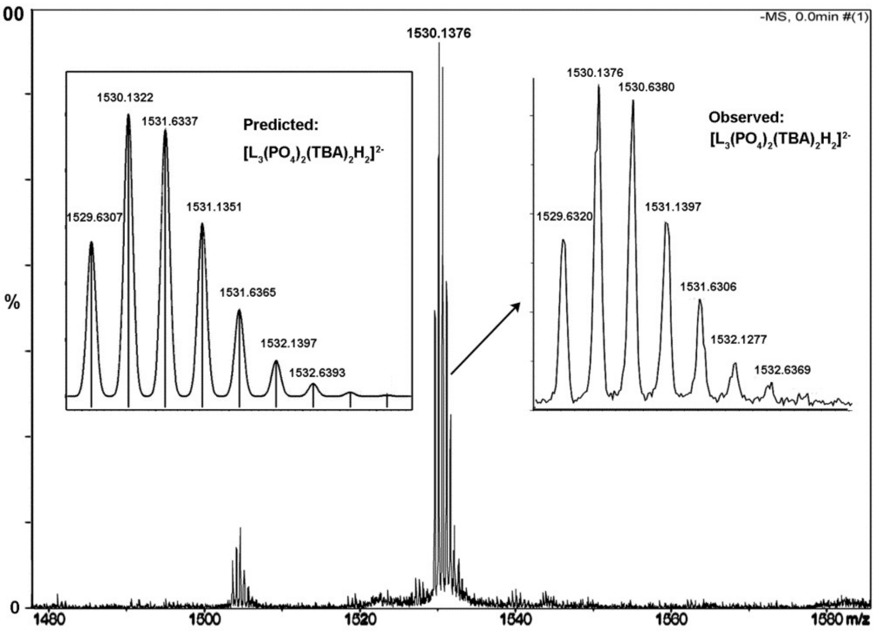

Supplementary Figure 2. HR-ESI-QTOF mass spectrum of host **2**.

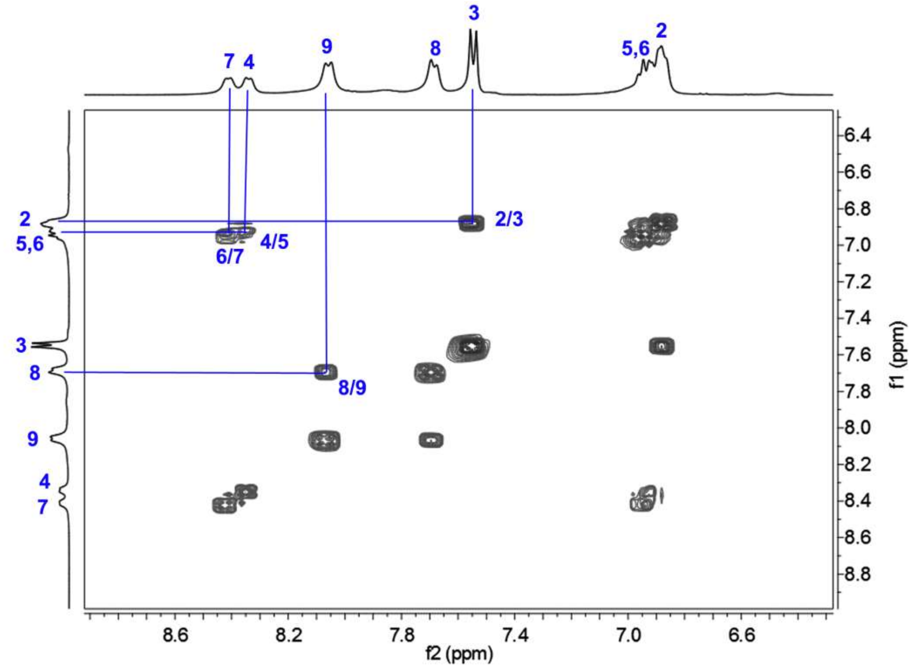

Supplementary Figure 3. Selected part of ^{1}H-^{1}H COSY (correlation spectroscopy) spectra (400 MHz, acetone-d_6/1.5% H_2O, 20 °C) of complex **2** $(TBA)_6[(PO_4)_2(\mathbf{L})_3]$ in the presence of 1 equiv. of Ch•Cl.

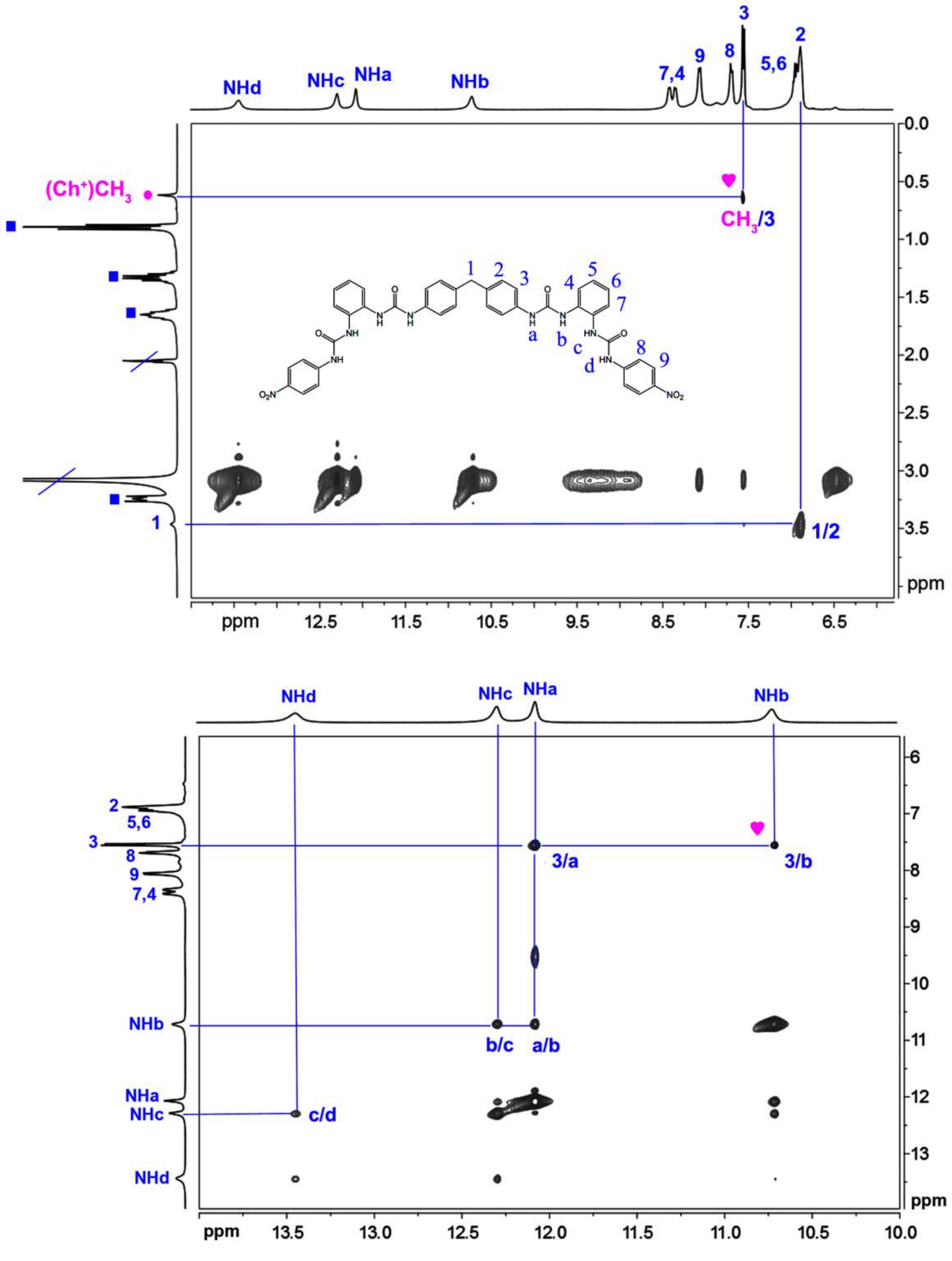

Supplementary Figure 4. Selected part of ^1H-^1H NOESY spectra (400 MHz, acetone-d_6/1.5% H_2O, 20 °C) of complex **2** $(TBA)_6[(PO_4)_2(\mathbf{L})_3]$ in the presence of 1 equiv. of Ch•Cl (TBA$^+$, ■).

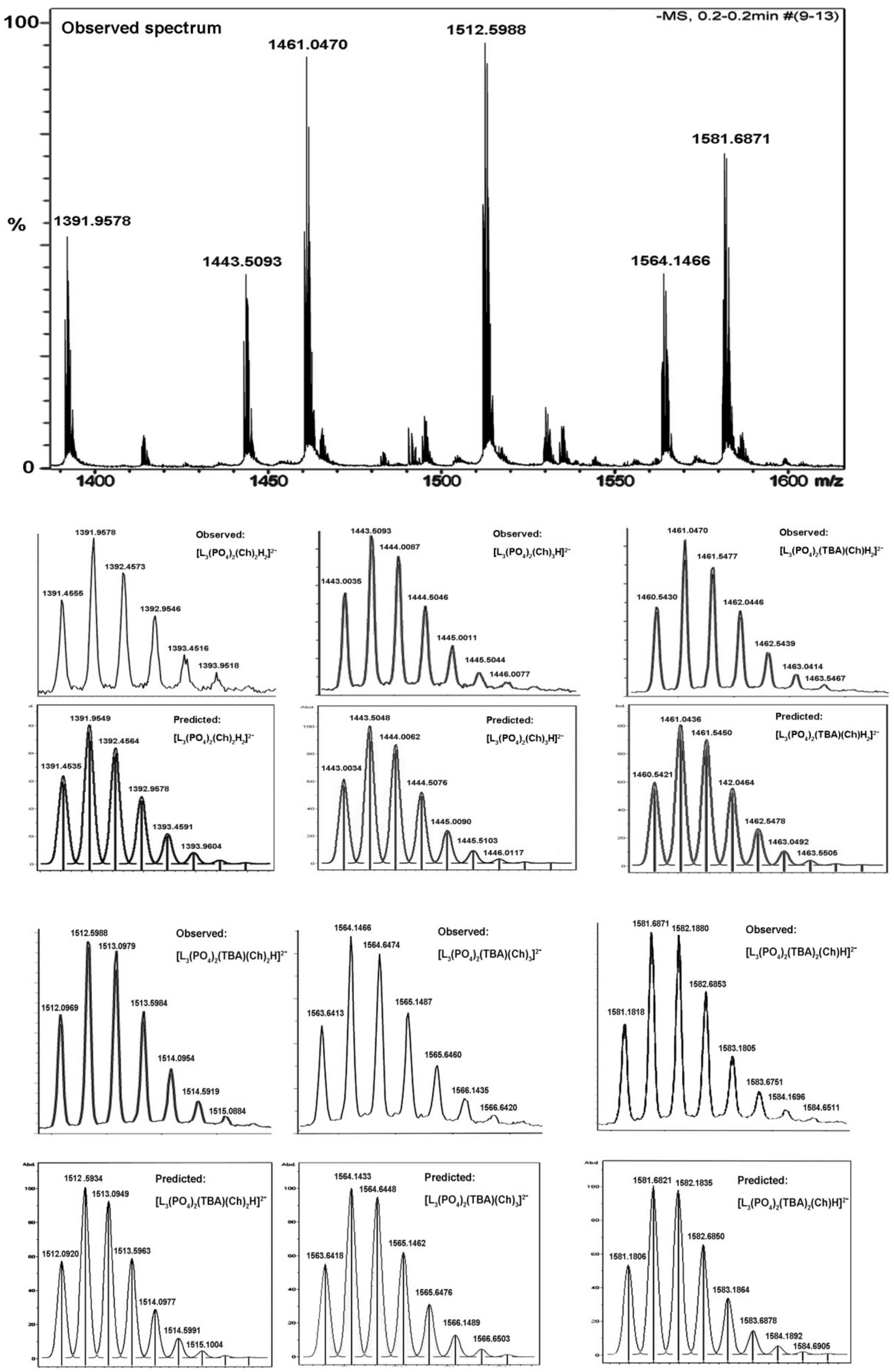

Supplementary Figure 5. HR-ESI-QTOF mass spectrum of host **2**/1 equiv. Ch•Cl, confirming the identity of the host-guest assembly.

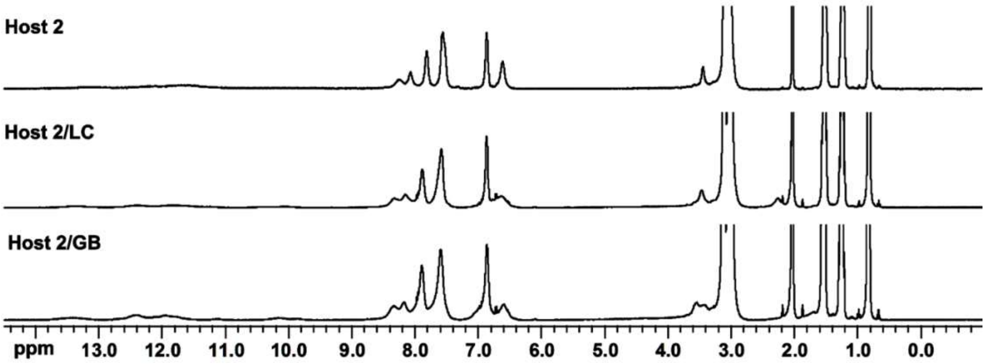

Supplementary Figure 6. Stacking ^{1}H NMR (acetone-d_6/1.5% D_2O, 400 MHz) spectra of host **2** alone and in the presence of 1 equiv. of LC, GB, showing that no significant changes were induced by LC or GB.

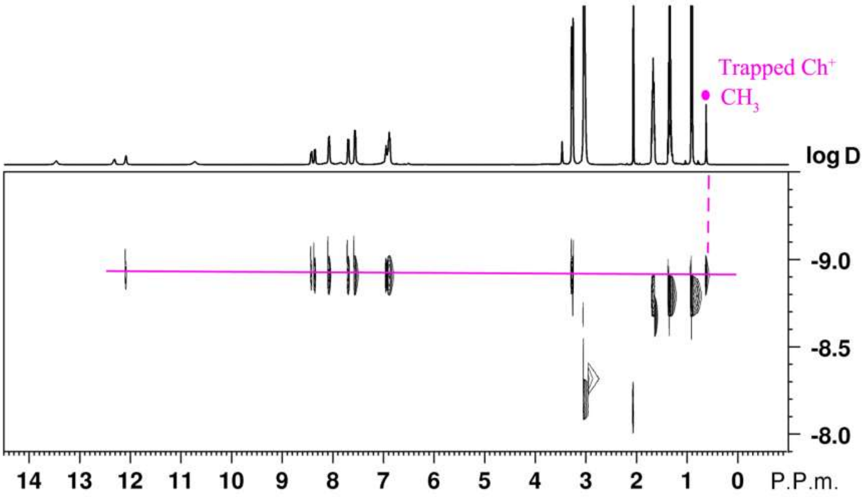

Supplementary Figure 7. ^{1}H DOSY NMR (acetone-d_6/1.5% H_2O, 500 MHz) spectrum of $Ch^{+}\subset$ **2** at 293 K.

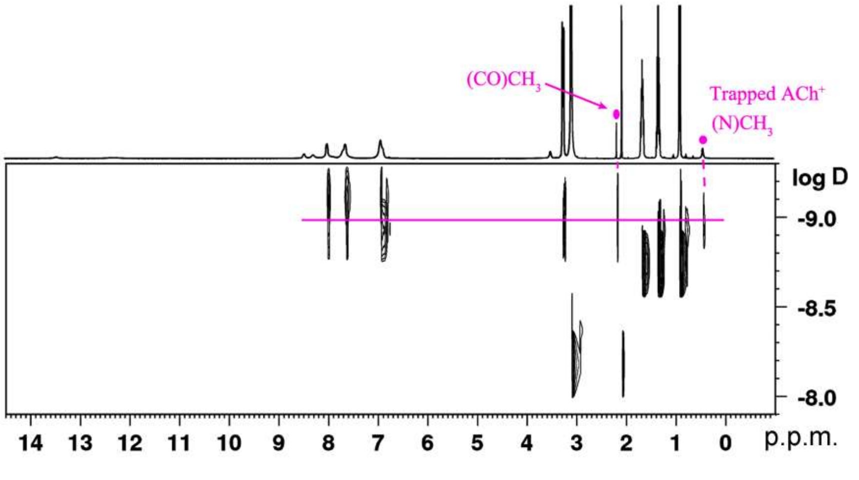

Supplementary Figure 8. ^{1}H DOSY NMR (acetone-d_6/1.5% H_2O, 500 MHz) spectrum of $ACh^{+}\subset$ **2** at 293 K.

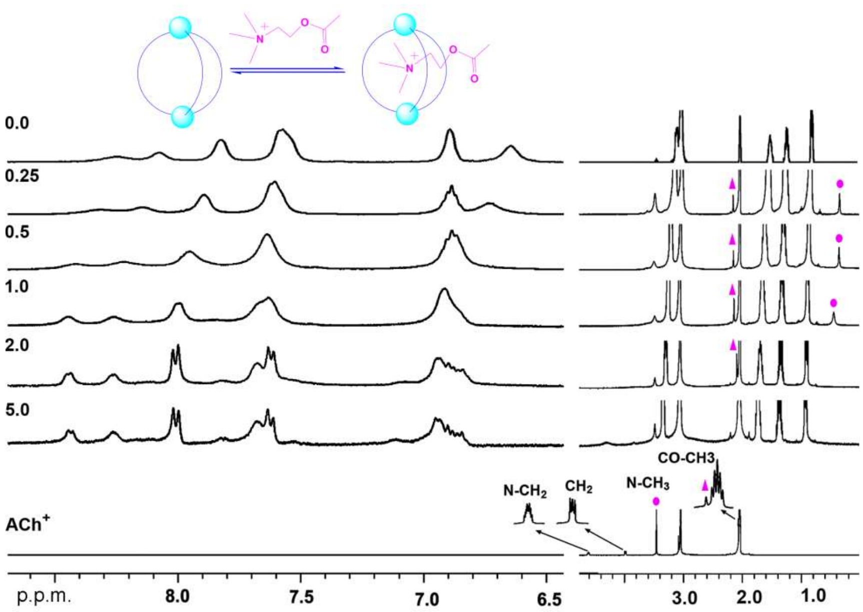

Supplementary Figure 9. Stacking ^{1}H NMR (acetone-d_6/1.5% D_2O, 400 MHz) spectra of complex **2** $(TBA)_6[(PO_4)_2(L)_3]$ alone (0.0) and in the presence of equivalents (labeled by numbers) of ACh^+•Cl, and the spectrum of free ACh•Cl (ACh^+, • ($N-CH_3$) and ▲ ($COCH_2$) respectively).

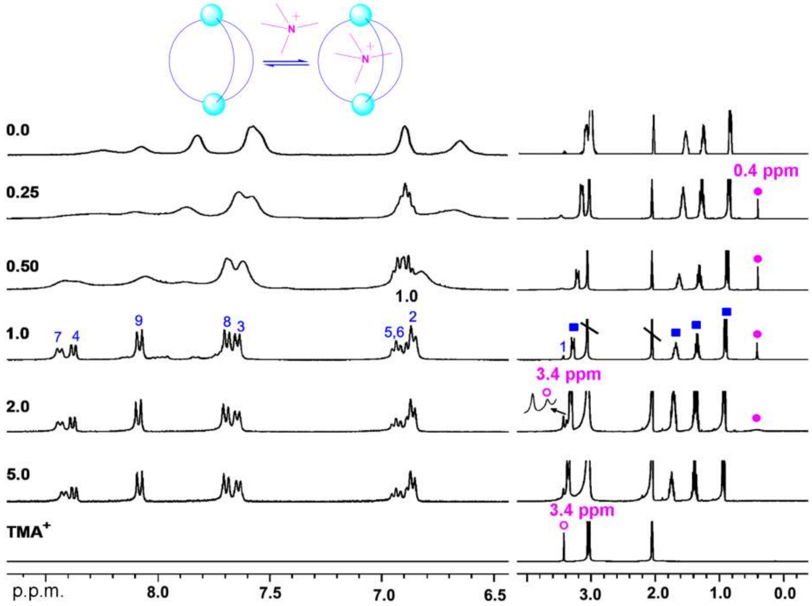

Supplementary Figure 10. Stacking ^{1}H NMR (acetone-d_6/1.5% D_2O, 400 MHz) spectra of complex (host) **2** in the presence of TMA•Cl (equivalents are labeled by numbers on the left side), where signals of the free and trapped TMA^+ are marked with ○ and • respectively, and TBA^+ protons are marked with ■.

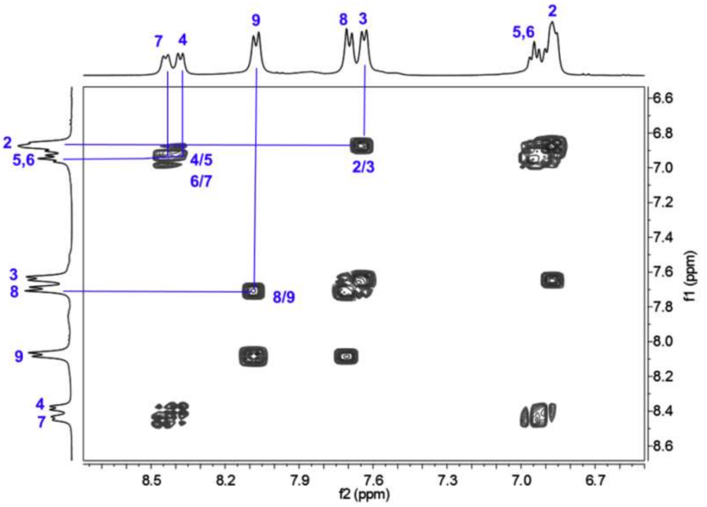

Supplementary Figure 11. Selected part of ^{1}H-^{1}H COSY (400 MHz, acetone-d_6/1.5% H_2O, 20 °C) spectrum of complex **2** $(TBA)_6[(PO_4)_2(\mathbf{L^2})_3]$ in the presence of 1 equiv. of TMA•Cl.

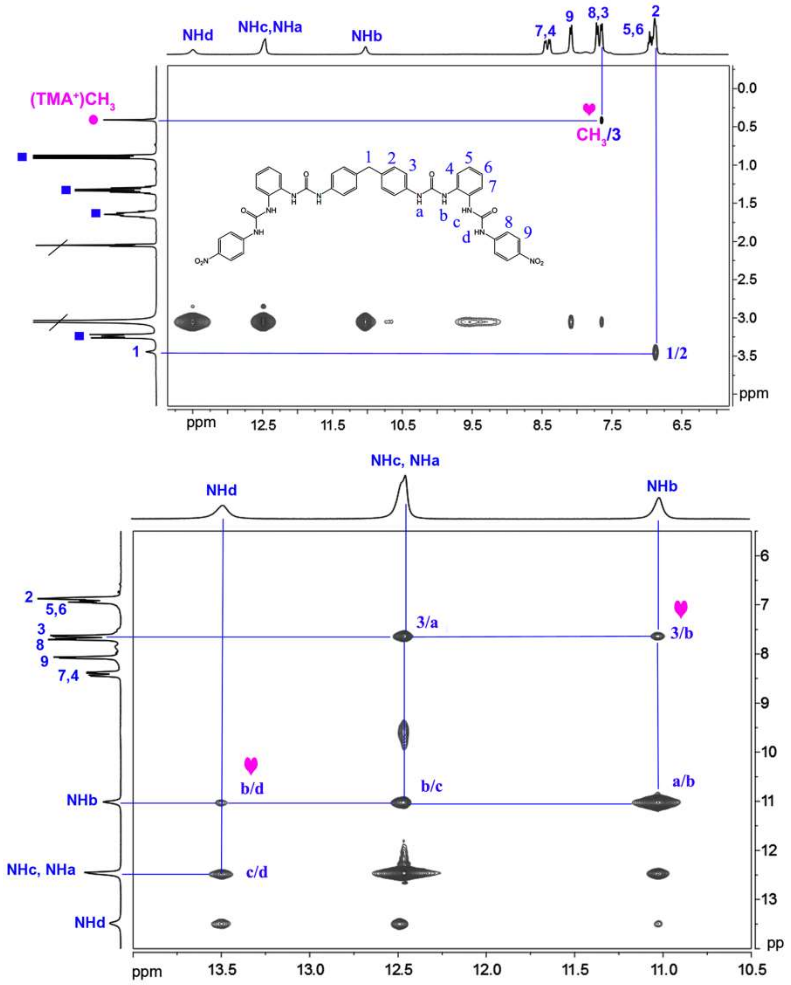

Supplementary Figure 12. Selected part of ^{1}H-^{1}H NOESY (400 MHz, acetone-d_6/1.5% H_2O, 20 °C) spectra of complex **2** $(TBA)_6[(PO_4)_2(\mathbf{L})_3]$ in the presence of 1 equiv. of TMA•Cl (TBA^+, ■).

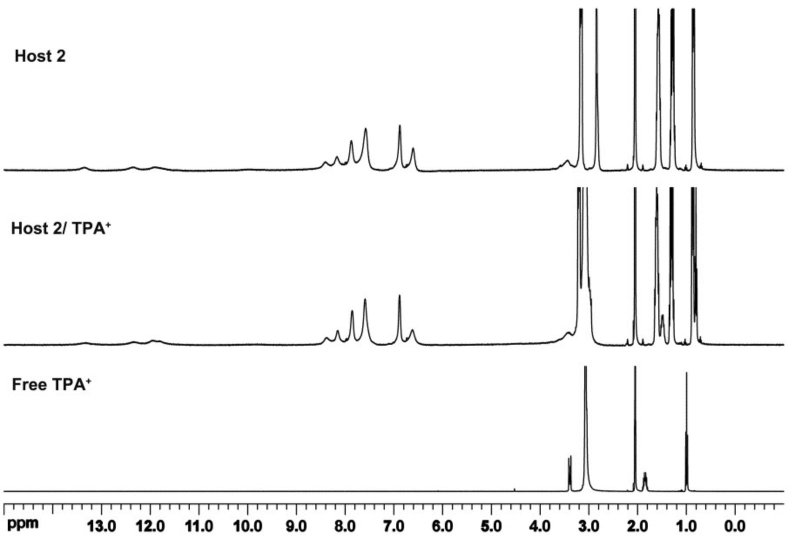

Supplementary Figure 13. Stacking ^{1}H NMR (acetone-d_6/1.5% D_2O, 400 MHz) spectra of complex (host) **2** alone and in the presence of 1 equivalents of TPA•Cl.

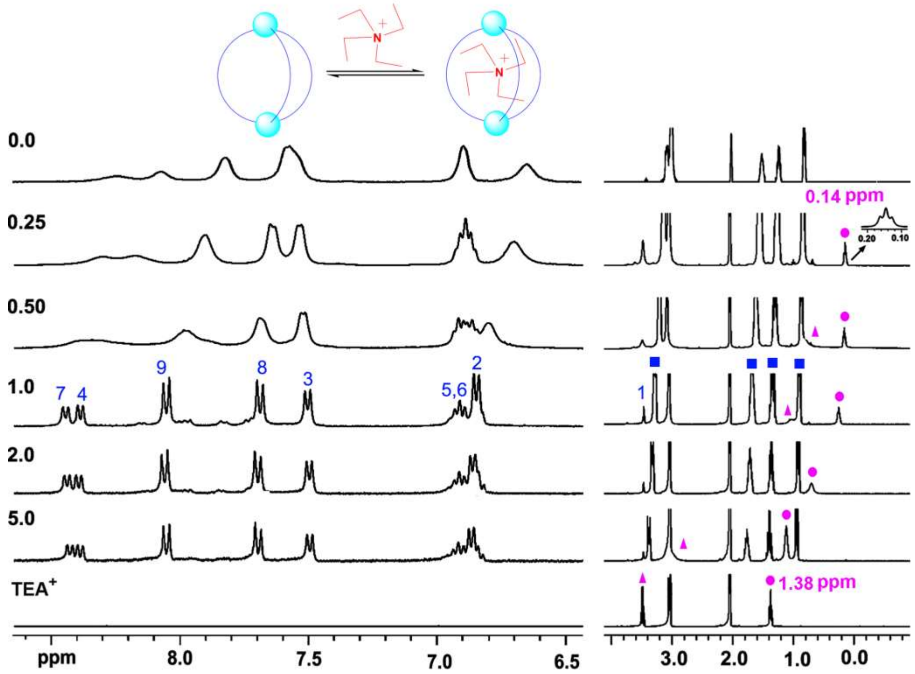

Supplementary Figure 14. Stacking ^{1}H NMR (acetone-d_6/1.5% D_2O, 400 MHz) spectra of complex **2** in the presence of different equivalents (as indicated by numbers on the left side) of TEA•Cl, where signals of the TEA$^+$ are marked with • (Me) and ▲ (NCH_2) respectively, and TBA$^+$, ■.

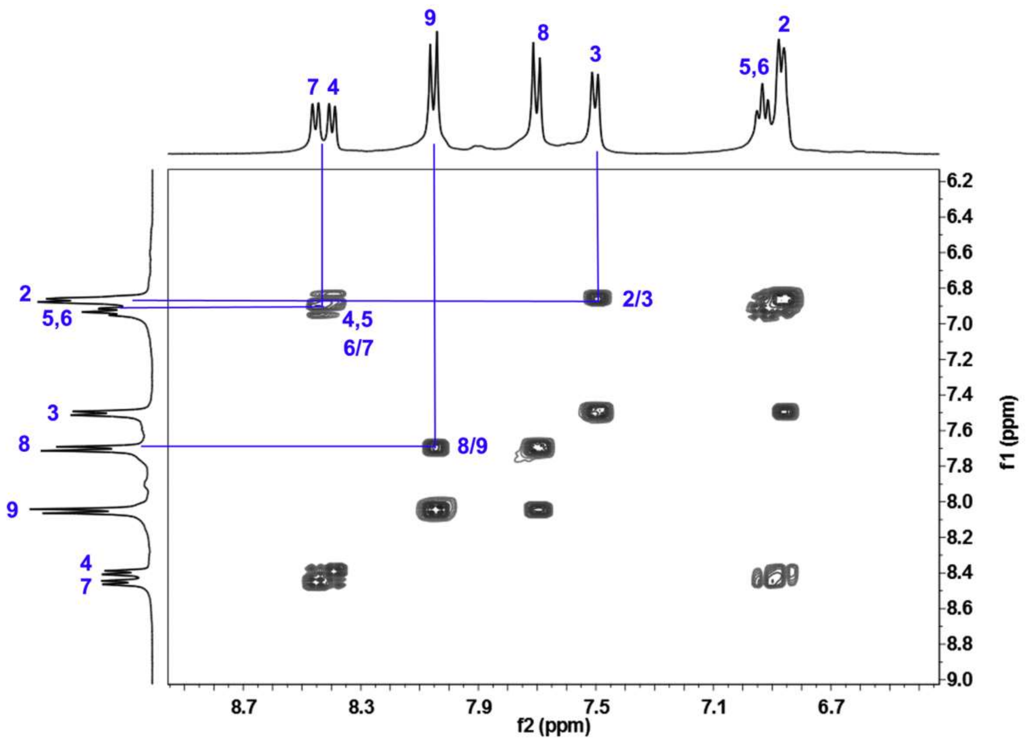

Supplementary Figure 15. Selected part of ^1H-^1H COSY (400 MHz, acetone-d_6/1.5% H_2O, 20 °C) spectrum of complex **2** $(TBA)_6[(PO_4)_2(\mathbf{L^2})_3]$ in the presence of 1 equiv. of TEA•Cl.

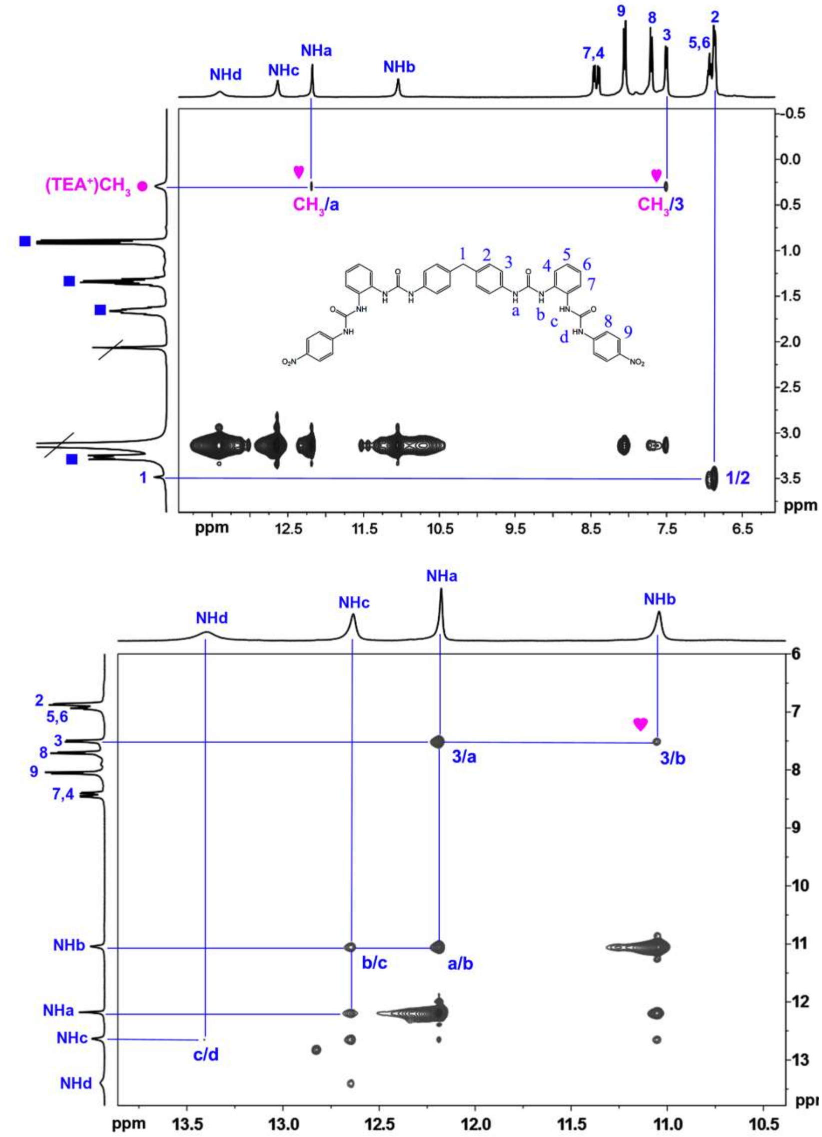

Supplementary Figure 16. Selected part of ^{1}H-^{1}H NOESY (400 MHz, acetone-d_6/1.5% H_2O, 20 °C) spectra of complex **2** $(TBA)_6[(PO_4)_2(\mathbf{L^2})_3]$ in the presence of 1 equiv. of TEA•Cl (TBA^+ are marked with ■).

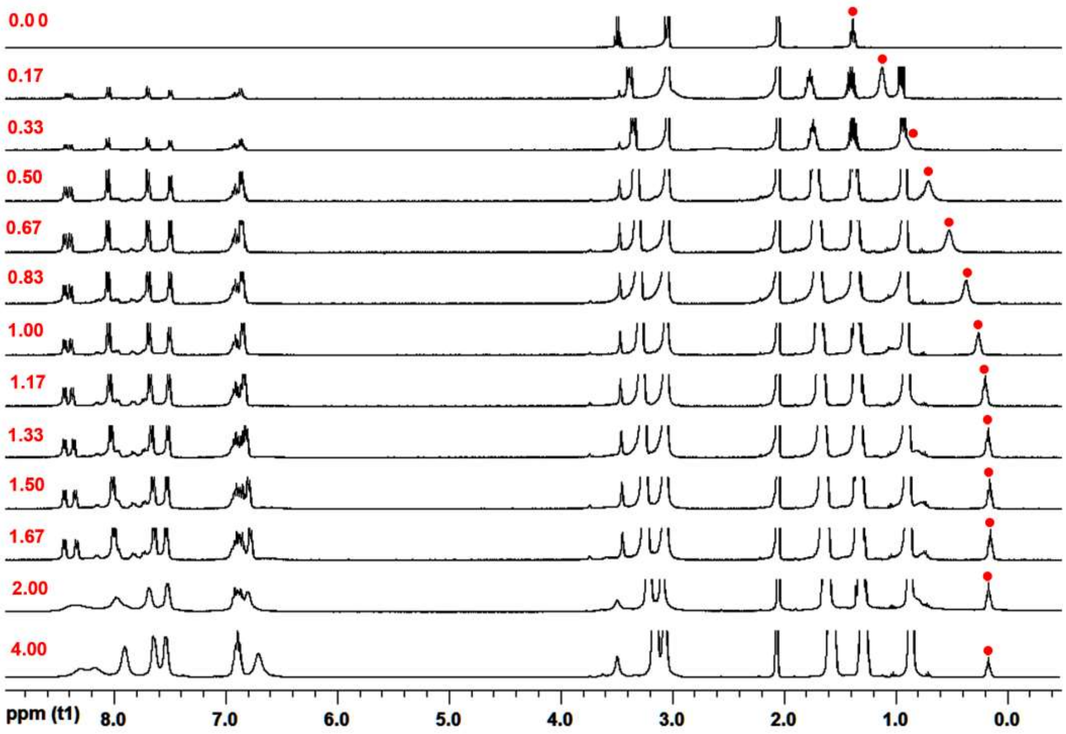

Supplementary Figure 17. Stacking ^{1}H NMR (acetone-d_6/1.5% D_2O, 400 MHz) spectra of TEA•Cl before (0.0) and after addition of equivalents of complex **2** $(TBA)_6[(PO_4)_2(\mathbf{L})_3]$ (numbers on the left side indicate equivalents of complex **2**), where signals of (TEA^+) CH_3 are marked with are marked with •.

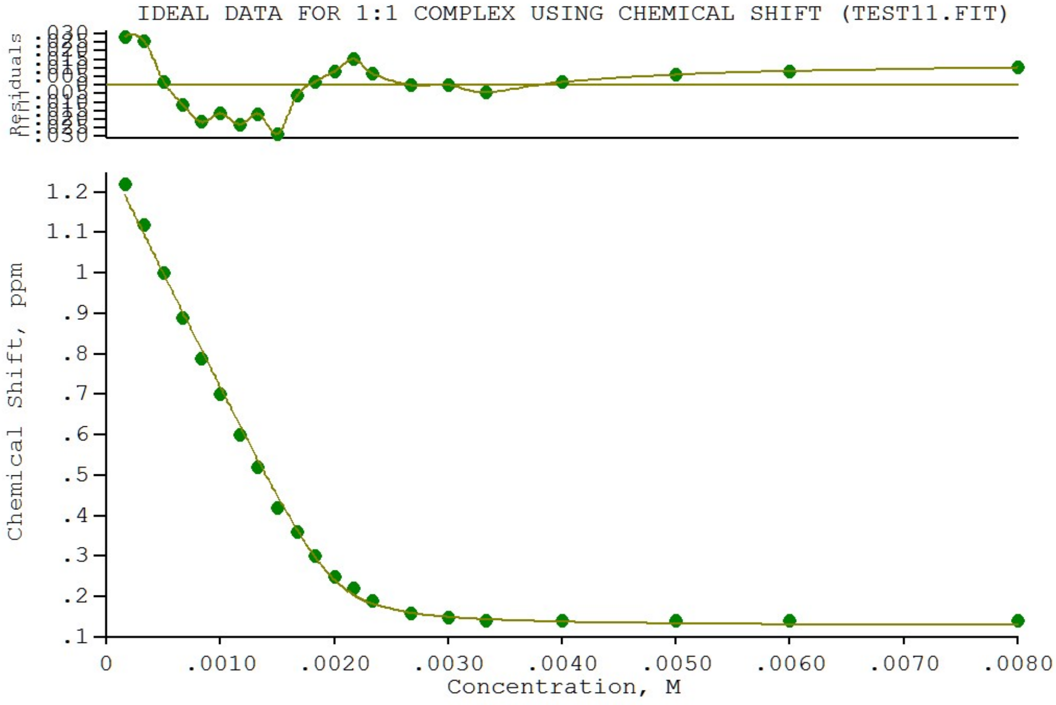

Supplementary Figure 18. Fitting results of the shifts profile of (TEA^+)CH_3 to a 1:1 mode with WinEQNMR[1]. Association constants: TEA^+ with complex **2:** $K_{(TEA+)}$ = 4.4381 x 10^4 M^{-1}, Max error = 2.8%.

Supplementary Table 3. Selectivity of complex of **2** to guest ions obtained by ^{1}H NMR competition experiments with TEA^+ as the reference in acetone-d_6/1.5% H_2O at 293 K.

Guest	$\delta_{observed}$ of TEA^+ (ppm)	$K(G)/K(TEA^+)$
**TMA^+**	1.14	17.4
**Ch^+**	1.17	24.1
**Ach^+**	0.79	1.2
**LC, GB**	0	<<1
Selectivity: $K(Ch^+)/K(ACh^+) = 20$		

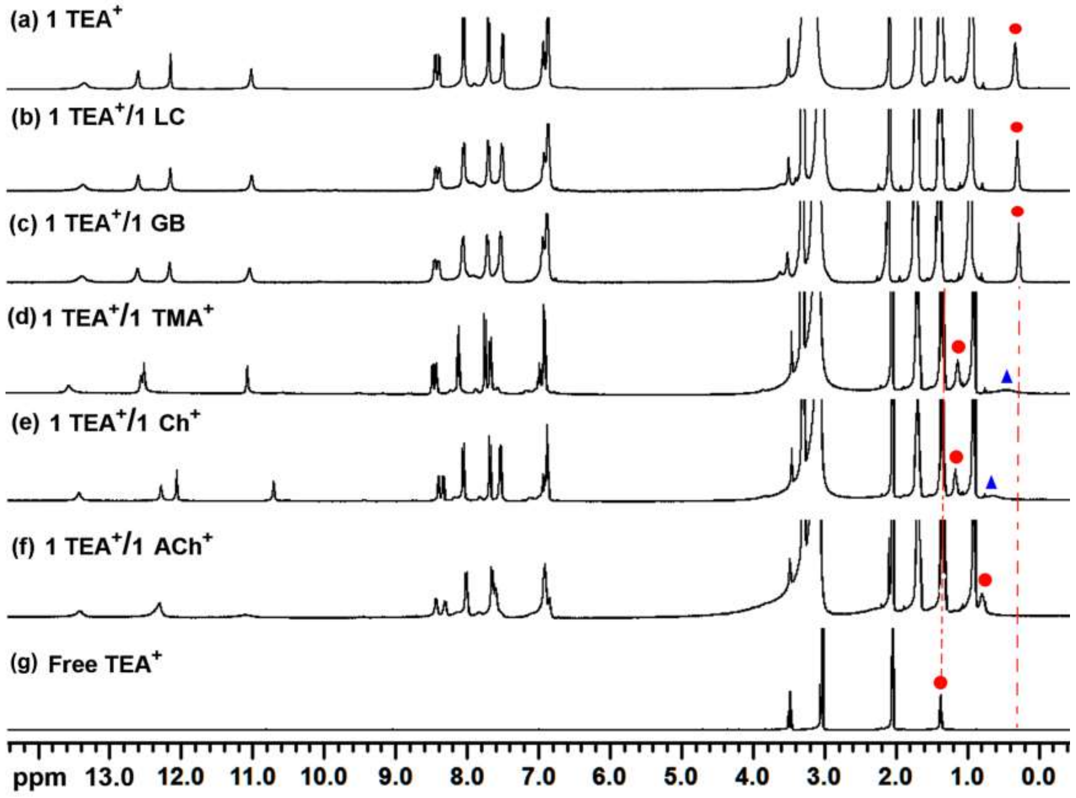

Supplementary Figure 19. Stacking ^{1}H NMR (acetone-d_6/1.5% H_2O, 400 MHz) spectra of complex **2** $(TBA)_6[(PO_4)_2(\mathbf{L})_3]$ in the presence of 1 equiv. of (a) TEA•Cl, and in the coexistence of 1 equiv. of (b) LC, (c) GB, (d) TMA•Cl, (e) Ch•Cl, (f) ACh•Cl and (g) the spectrum of free TEA•Cl, showing TMA^+, Ch^+ and Ach^+ induced strong competition with TEA^+ but not LC and GB (● CH_3-TEA^+; ▲ CH_3-Ch^+ or CH_3-TMA^+).

Supplementary Table 4 Chemical shifts of protons of SP^+ upon complexation with host **2**.

	H1	H2	H3	H4	H5	H6	H7	H8
SP^+	4.42	8.76	8.12	7.94	7.21	7.63	6.81	3.07
SP^+/Host **2**	2.29	7.71	7.43	7.47	6.68	7.22	6.62	2.99
$\Delta\delta$	-2.13	-1.05	-0.69	-0.47	-0.53	-0.41	-0.19	-0.08

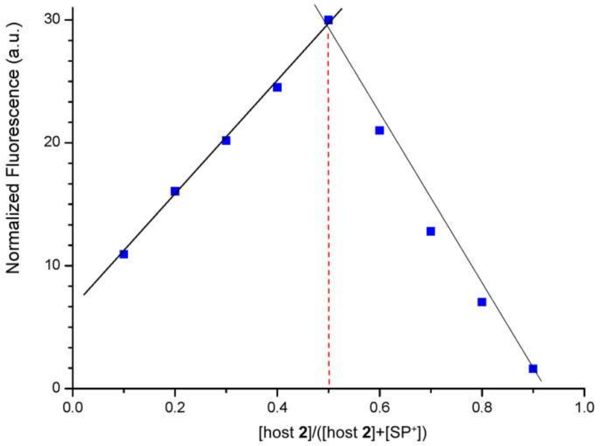

Supplementary Figure 20. Job's plot analyses for the host **2** with SP•I in acetone/1.5% H_2O, λ_{ex} = 470 nm, λ_{em} = 575 nm, [host **2**] + [SP•I] = 100 μM.

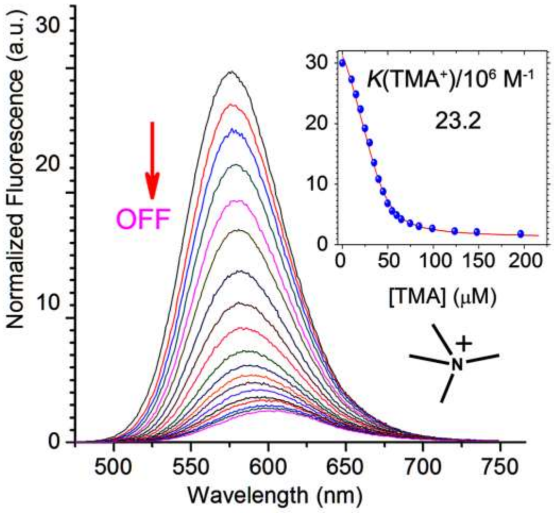

Supplementary Figure 21. Emission spectra of SP^+/host 2 (50 μM/50 μM) before and after addition aliquot equiv. of TMA•Cl, showing a fluorescence "switch off" response. All spectra were recorded in acetone/1.5% H_2O with λ_{ex} = 470 nm. Insets show structures of analytes and the corresponding association constants determined by fitting the titration curves at λ_{em} = 575 nm (blue dots) to a 1:1 (host : guest) binding mode by the Dynafit program (error < 10%)[2].

Supplementary Table 5. Selectivity of host **2** relative to TEA^+, or based on association constants, for 1:1 complexes with analytes in acetone/1.5% H_2O at 298 K.

	SP^+	TEA^+	TMA^+	Ch^+	Ach^+	GB	LC
$^aK_{ass}$(guest)/K_{ass}(TEA^+)	–	1.0	17.4	24.1	1.2	<<1	<<1
$^bK_{ass}$(guest)/K_{ass}(TEA^+)	–	1.0	11.4	16.3	1.1	0..01	0.02
$^bK_{ass}$(guest) (10^6 M^{-1})	1.15	2.04	23.2	33.3	2.22	0.021	0.035
error%	9.9	2.9	8.3	9.9	3.8	6.4	7.5
Summary:	$^aK_{ass}(Ch^+)/K_{ass}(ACh^+) = 20$			$^bK_{ass}(Ch^+)/K_{ass}(ACh^+) = 15$			
	$^aK_{ass}(TMA^+)/K_{ass}(ACh^+) = 0.7$			$^bK_{ass}(TMA^+)/K_{ass}(ACh^+) = 0.7$			

[a]Data determined by ^{1}H NMR competition titrations with TEA^+ as the reference.

[b]Data determined by fluorescence displace method based on a host **2**-SP^+ dye complex.

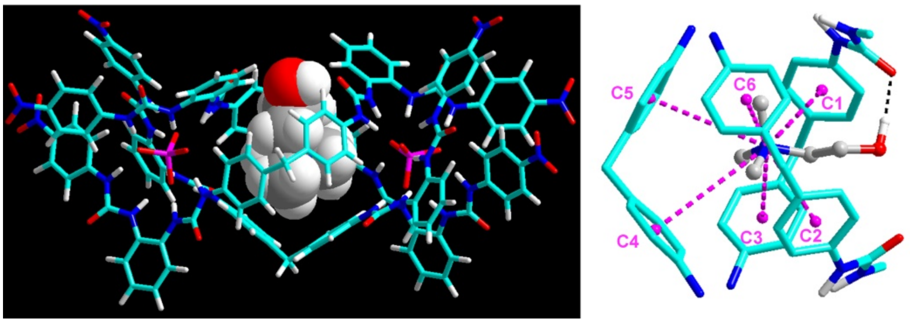

N-C1	N-C2	N-C3	N-C4	N-C5	N-C6	Average
4.3	4.9	4.8	4.8	4.5	4.7	4.7

Supplementary Figure 22. DFT optimized structure of Ch^+⊂**2** (left) and choline binding sites (right) with data of N···centroid distances for evaluating the cation-π interactions (purple dashed lines [Å]).

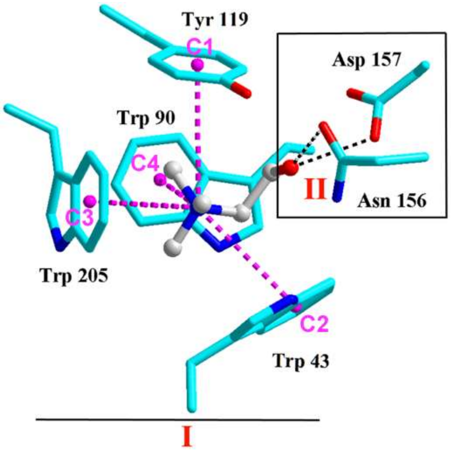

N-C1	N-C2	N-C3	N-C4	Average
4.4	4.2	4.5	4.2	4.3

Supplementary Figure 23. Binding sites in crystal structure of Ch^+⊂ChoX with data of N···centroid distances for evaluating the cation-π interactions (purple dashed lines [Å]).

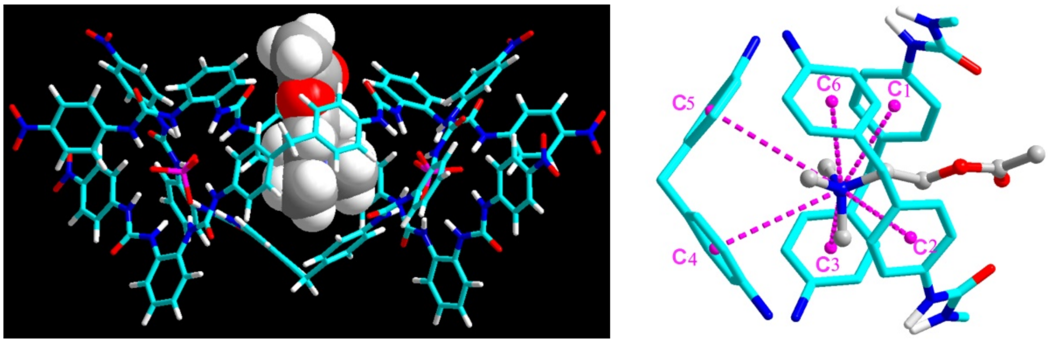

N-C1	N-C2	N-C3	N-C4	N-C5	N-C6	Average
4.3	4.8	4.7	4.9	4.5	4.5	4.6

Supplementary Figure 24. DFT optimized structure of $ACh^{+}\subset$**2** (left) and choline binding sites (right) with data of N···centroid distances for evaluating the cation-π interactions (purple dashed lines [Å]).

Supplementary Table 6. DFT calculated results regarding host-guest binding.

	ΔE_{int}	E_{def}(guest)	$E_{def}([(PO_4)_2(\mathbf{L}^2)_3])$	ΔG	$-T\Delta S$	ΔH
Ch^+	-46.3	0.9	4.6	-39.4	1.5	-40.9
ACh^+	-45.0	0.3	3.5	-36.6	4.6	-41.2
ΔE_{int} decomposition						
	ΔE_{disp} (2-body)		$\Delta E_{electro}$		ΔE_{disp} (3-body)	
Ch^+	-43.3		-270.5		1.5	
ACh^+	-49.6		-260.8		1.6	

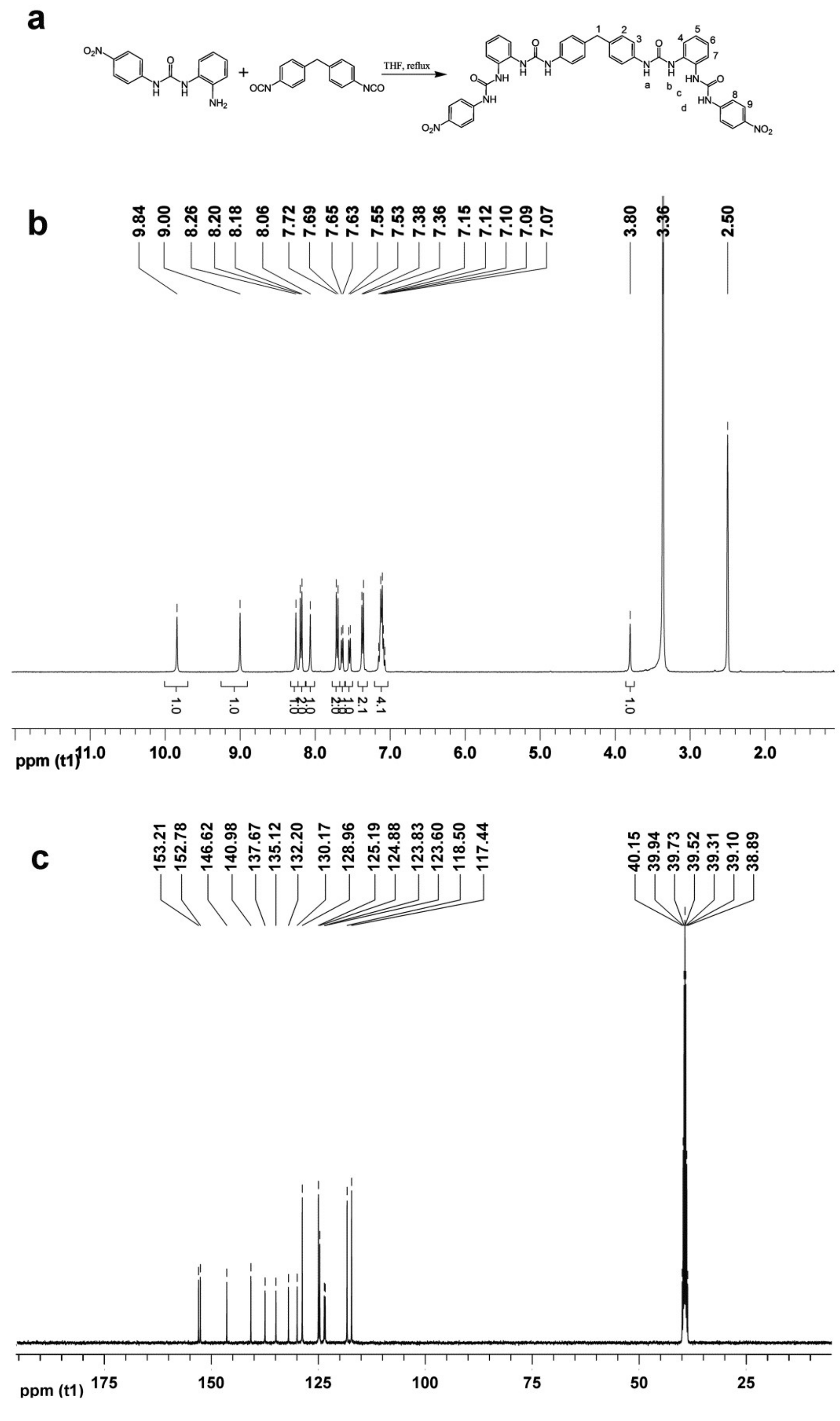

Supplementary Figure 25. (**a**) Scheme showing the synthesis of ligand $\mathbf{L^2}$. (**b**) ^{1}H and (**c**) ^{13}C NMR spectra of $\mathbf{L^2}$.

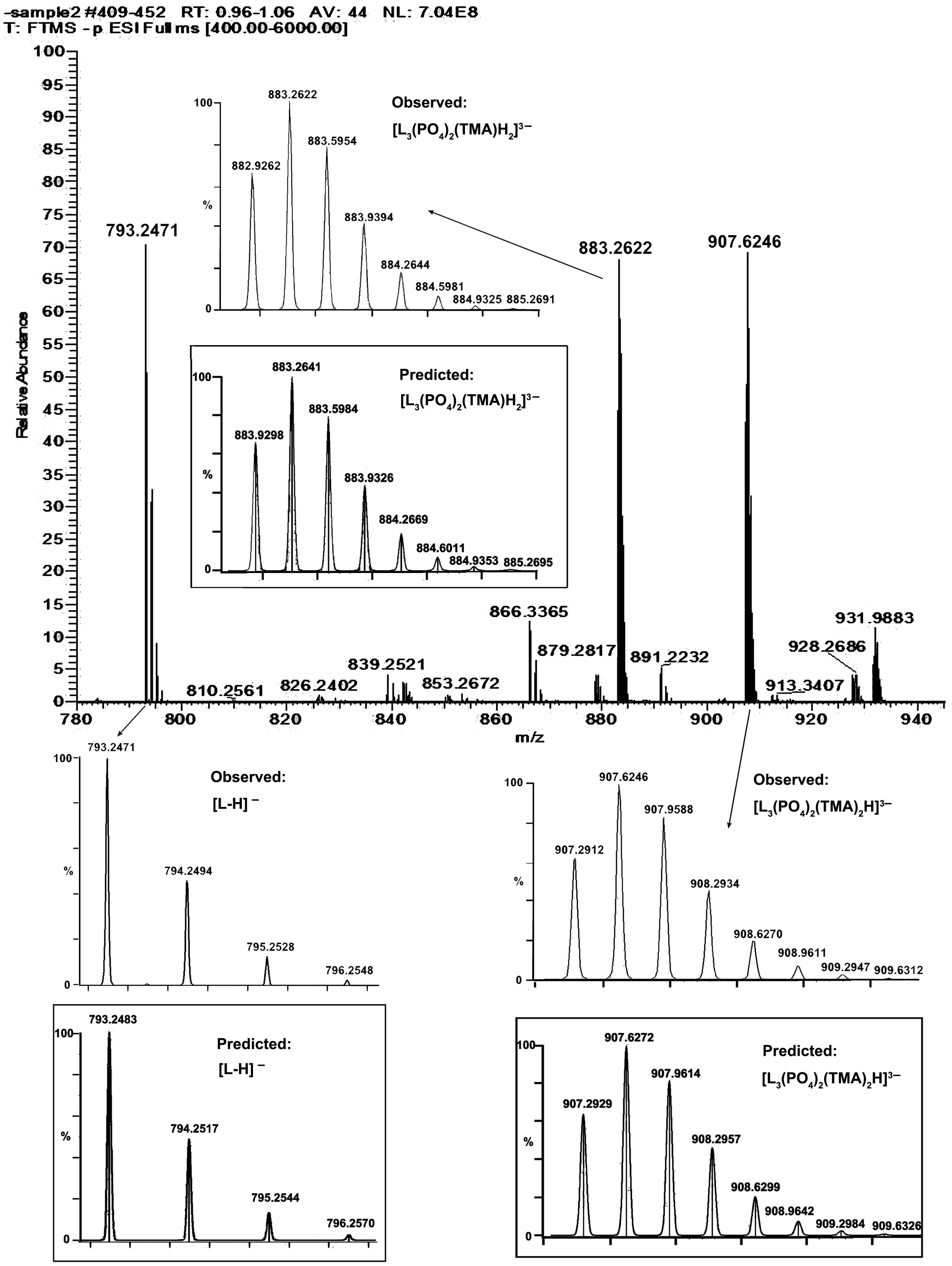

Supplementary Figure 26. HR-ESI-QTOF mass spectrum of host **1**.

Supplementary methods

The binding constant of TEA^+ based on ^1H NMR titrations

Calculations by WinEQNMR2 Version 2.00 by Michael J. Hynes

Program run at 17:54:54 on 07/21/2016

IDEAL DATA FOR 1:1 COMPLEX USING CHEMICAL SHIFT (TEST11.FIT)

Reaction: G + H = HG

FILE: TEST11.FIT

IDEAL DATA: K1 = 1000; DELTA G = 1.38; DELTA HG = 0.14

File prepared by C. Jia, July 21 2016

Equilibrium constants are floating point numbers

NO.	A	PARAMETER	DELTA	ERROR	CONDITION	DESCRIPTION
1	1	4.43813E+04	1.000E-01	9.503E+03	2.887E+00	K1
2	1	1.28312E+00	1.000E-02	8.895E-03	1.141E+00	SHIFT G
3	1	1.25376E-01	1.000E-03	7.731E-03	2.977E+00	SHIFT HG

0RMS ERROR = 1.59E-02 MAX ERROR = 2.84E-02 AT OBS.NO. 1

RESIDUALS SQUARED = 4.56E-03

RFACTOR = 2.5573 PERCENT

Binding constants based on ^1H NMR competition

Association constants of $TMA^{+,}$ CH^+ and Ach^+ (labeled as G) with host **2** (labeled as H) were estimated by ^1H NMR competition experiments according to the following equations:

$[H] + [TEA^+] = [H\bullet\ TEA^+]$, $\quad K(TEA^+) = [H\bullet\ TEA^+]/([H][TEA^+])$

$[H] + [G] = [H\bullet\ G]$, $\quad K(G) = [H\bullet\ G]/ ([H][G])$

$K(G)/ K(TEA^+) = ([H\bullet\ G]\ [TEA^+])/([H\bullet\ TEA^+][\ G])$ (1)

Given the fact that two equivalents of any one of the test guests is capable of binding host **2** to saturation, it is thus assumed that in the coexistence of one equiv. of TEA^+ and one other tested guest, host **2** is completely bound and gives an equation as:

$[H\bullet\ G] + [H\bullet\ TEA^+] = [H]_{all} = C_0$

Since equally one equiv. of TEA^+ and one guest were added, so

$[H]_{all} = [TEA^+]_{all} = [G]_{all} = C_0$, then

$[H\bullet\ G] + [H\bullet\ TEA^+] = [TEA^+] + [H\bullet\ TEA^+] = [G] + [H\bullet\ G] = C_0$ (2)

So

$[H\bullet\ G] = [TEA^+], [H\bullet\ TEA^+] = [G]$ (3)

From eq. (1), (2) and (3),

$K(G)/ K(TEA^+) = \{ [TEA^+]/(C_0 - [TEA^+])\}^2$ (4)

On the other hand, given the strong binding ($K_{(TEA+)} > 10^4\ M^{-1}$) and fast exchange features of TEA^+ binding, the observed resonance of TEA^+ was calculated as:

$\delta_{observed} = \delta_{free}X_{free} + \delta_{bound}\,X_{bound}$

where $X_{free} = [TEA^+]/ C_0$, $X_{bound} = [H\bullet\ TEA^+]/ C_0$.

In former experiments, $\delta_{free,}$ δ_{bound} were determined as 1.38 ppm and 0.14 ppm respectively (Supplementary Figure 14), so

$\delta_{observed} = 1.38[TEA^+]/ C_0 + 0.14\ [H\bullet\ TEA^+]/ C_0$ (5)

From eq. (2) and (5),

$[TEA^+] = C_0 (\delta_{observed} -0.14)/1.24$ (6)

From eq. (4) and (6),

$K(G)/ K(TEA^+) = \{[(\delta_{observed} -0.14)/1.24]/[(1-(\delta_{observed} -0.14)/1.24)]\}^2$ (7)

Binding constants based on fluorescence titrations

The script file for SP^+-Host **2** fitting:

"Host (H) is varied while a fluorescently labeled guest (Dye, D*) is held constant. Fit fluorescence increase to 1:1 binding model.

```
;____________________________________________________________
[task]
   data = equilibrium
   task = fit
[mechanism]
   HD* <==> H + D*     :     Ka     association
[constants]
   Ka = 1.15 ?
[concentrations]
   D* = 50    ; Corresponding to K = xx vM-1
[responses]
   D*     = 0.02
   HD* = 0.7075
[data]
   variable        H
   set              D*
[output]
directory ./JCDoutput/fit01-SP-H
[set:D*]
H, vM              fluorescence
0                  1.00000
4.97512            3.17492
9.90099            6.92613
14.77833   10.37756
19.60784           13.38882
24.39024   17.2851
29.12621   20.06195
33.81643   23.57678
38.46154   25.87335
43.0622            28.06084
47.61905   29.97763
52.1327            32.00295
56.60377   33.0388
65.42056   33.69139
78.34101   34.66063
90.90909   34.80015
166.66667  35.10092
230.76923  35.10142
[end]"
```

The script file for Ch^+-Host **2**/SP^+ fitting:

"Fit the competition experiment (unlabeled competitor G vs labeled dye D*)

while treating KD* as a fixed parameter.

```
;______________________________________________________________________
[task]
task = fit
data = equilibria
[mechanism]
```

```
H.D* <==> H + D*    : KD* association
H.G    <==> H + G      : KG      association
[constants]
KD* = 1.15
KG    = 33.3 ?
[concentrations]
D* = 50    ; vM
H    = 50    ; vM
[responses]
D* = 0.02 ;reponse= I per vM of D*
H.D* = 0.7075 ;reponse= I per vM of H.D*
[data]
variable G
set            D*
[output]
directory ./JCDoutput/Ch-fit02

[set:D*]
G,vM                fluorescence
0               30
9.995         26.61819
19.98002    21.65997
29.95507    15.53198
39.92016    9.97362
49.87531    5.82671
59.82054    4.28001
69.75585    3.31202
84.64028    2.78554
99.50249    2.32691
124.2236    2.02193
148.88337  1.82682
198.0198    1.5801
[end]”
```

Density Functional Theory (DFT) molecular modeling

The Density Functional Theory (DFT) method, augmented with empirical dispersion term (D3), has been utilized for systems under study[3]. In previous works, two of the authors, Jiří Hostaš and Pavel Hobza have demonstrated that DFT-D3 method is reliable enough for calculations of both the ΔG relative estimates for host-guest systems as well as conformational stabilities of flexible polymethylene-bridged heteroaromatic dimers[4,5]. The B-LYP functional combined with def2-SVP and def2-TZVPP basis sets was chosen for geometry optimization and interaction energy (ΔE_{int}) calculations, respectively[6,7]. All calculations have been performed utilizing COSMO solvation model in order to include effect of the environment (acetone)[8]. All DFT calculations were performed in Turbomole 6.6 software[9]. The second derivatives and entropy analysis were calculated with Amber at 298 K[10]. The ‘cuby framework’ was used to automate the calculations[11]. As the X-ray crystal structures of acetylcholine and choline molecules with triple anion helicates were not available, the starting geometries for DFT-D geometry optimizations were prepared by modification of the crystal structure of the complex with TMA^+ moiety. To

understand the nature of the stability of the complexes, the dispersion energy (ΔE_{disp}), deformation energies {E_{def}(guest) and $E_{def}([(PO_4)_2(\mathbf{L}^2)_3]$} and electrostatic energies ($\Delta E_{electro}$) were calculated. The $\Delta E_{electro}$ was determined by the Coulomb law calculated by using natural bond orbital atomic charges. It has been shown that these crude estimates of $\Delta E_{electro}$ correlate well with more advanced Energy Decomposition Analysis method[4].

Supplementary References

1. Hynes, M.J. EQNMR: a computer program for the calculation of stability constants from nuclear magnetic resonance chemical shift data. *J. Chem. Soc., Dalton Trans.*, 311-312 (1993).
2. Kuzmič, P. Program DYNAFIT for the analysis of enzyme kinetic data: Application to HIV proteinase. *Anal. Biochem.* **237**, 260-273 (1996).
3. Grimme, S., Antony, J., Ehrlich, S. & Krieg, H. A consistent and accurate ab initio parametrization of density functional dispersion correction (DFT-D) for the 94 elements H-Pu. *J. Chem. Phys.* **132**, 154104 (2010).
4. Hostaš, J. et al. A Nexus between Theory and Experiment: Non-Empirical Quantum Mechanical Computational Methodology Applied to Cucurbit[n]uril-Guest Binding Interactions. *Chem. Eur. J.* **22**, 17226-17238 (2016).
5. Rai, S.K. et al. Experimental and Theoretical Study for the Assessment of the Conformational Stability of Polymethylene-Bridged Heteroaromatic Dimers: A Case of Unprecedented Folding. *Cryst. Growth Des.* **16**, 1176-1180 (2016).
6. Lee, C., Yang, W. & Parr, R.G. Development of the Colle-Salvetti correlation-energy formula into a functional of the electron density. *Phys. Rev. B* **37**, 785-789 (1988).
7. Weigend, F. & Ahlrichs, R. Balanced basis sets of split valence, triple zeta valence and quadruple zeta valence quality for H to Rn: Design and assessment of accuracy. *Phys. Chem. Chem. Phys.* **7**, 3297-3305 (2005).
8. Klamt, A. & Schüürmann, G. COSMO: A New Approach to Dielectric Screening in Solvents with Explicit Expressions for the Screening Energy and its Gradient. *J. Chem. Soc., Perkin Trans. 2*, 799-805 (1993).
9. TURBOMOLE V6.6 2014, University of Karlsruhe and ForschungszentrumKarlsruhe GmbH, 1989 –2007, TURBOMOLE GmbH, since 2007; available from http://www.turbomole.com.
10. Case, D.A. et al. AMBER version 11, University of California, San Francisco (2010).
11. Řezáč, J. Cuby: An integrative framework for computational chemistry. *J. Comput. Chem.* **37**, 1230–1237 (2016).
