# Supplementary material for: Selective binding of choline by a phosphate-coordination-based triple helicate featuring an aromatic box
Source: Nat Commun. 2017 Oct 16;8:938. doi: 10.1038/s41467-017-00915-8 (PMC5643546; doi:10.1038/s41467-017-00915-8)
Supplement: Supplementary file 2 — Description of Additional Supplementary Information [file 41467_2017_915_MOESM2_ESM.pdf]

Description of Additional Supplementary Files

File Name: Supplementary Data 1
Description: Cartesian coordinates of the optimized geometries of $Ch^{+}\subset$**2** and $ACh^{+}\subset$**2**
