# Supplementary material for: Selective binding of choline by a phosphate-coordination-based triple helicate featuring an aromatic box
Source: Nat Commun. 2017 Oct 16;8:938. doi: 10.1038/s41467-017-00915-8 (PMC5643546; doi:10.1038/s41467-017-00915-8)
Supplement: Supplementary file 3 — Supplementary Data 1 [file 41467_2017_915_MOESM3_ESM.pdf]

Supplementary data-Cartesian coordinates

1# Caged Ch$^+$

310
N -0.022000000 8.137000000 24.261000000
C 0.773000000 9.409000000 24.015000000
H 0.113000000 10.276000000 24.182000000
H 1.141000000 9.419000000 22.977000000
H 1.621000000 9.444000000 24.718000000
C -1.120000000 8.015000000 23.217000000
H -1.727000000 7.125000000 23.441000000
H -0.677000000 7.931000000 22.214000000
H -1.751000000 8.916000000 23.266000000
C -0.663000000 8.208000000 25.637000000
H -1.214000000 7.271000000 25.819000000
H -1.350000000 9.068000000 25.665000000
H 0.128000000 8.330000000 26.393000000
C 0.896000000 6.900000000 24.227000000
C 1.741000000 6.725000000 22.944000000
H 1.550000000 6.970000000 25.116000000
H 0.235000000 6.027000000 24.363000000
H 2.566000000 7.458000000 22.920000000
H 1.123000000 6.909000000 22.037000000
O 2.316000000 5.430000000 22.924000000
H 1.632000000 4.805000000 22.558000000
C 10.677000000 8.047000000 32.472000000
C 10.521000000 6.774000000 31.881000000
C 9.681000000 9.038000000 32.317000000
N 11.878000000 8.345000000 33.246000000
C 4.252000000 2.915000000 27.810000000
C 3.825000000 3.194000000 26.497000000
C 4.850000000 3.927000000 28.581000000
H 4.106000000 1.914000000 28.244000000
C 4.002000000 4.477000000 25.952000000
H 3.335000000 2.413000000 25.895000000
C 4.631000000 5.499000000 26.707000000
H 3.650000000 4.709000000 24.940000000
C 5.054000000 5.221000000 28.047000000
N 4.811000000 6.806000000 26.188000000
C 5.061000000 7.116000000 24.850000000
N 5.121000000 8.497000000 24.640000000
O 5.200000000 6.268000000 23.956000000

C 4.744000000 9.165000000 23.462000000
C 4.680000000 8.554000000 22.181000000
C 4.334000000 10.521000000 23.580000000
C 4.112000000 9.251000000 21.100000000
H 5.028000000 7.523000000 22.065000000
C 3.612000000 10.566000000 21.233000000
H 4.032000000 8.738000000 20.129000000
C 3.775000000 11.202000000 22.489000000
C 2.821000000 11.225000000 20.102000000
H 3.429000000 12.236000000 22.638000000
C 9.378000000 6.481000000 31.135000000
H 11.312000000 6.025000000 32.006000000
H 4.409000000 11.010000000 24.559000000
C 1.323000000 10.908000000 20.177000000
H 3.218000000 10.879000000 19.126000000
H 2.961000000 12.324000000 20.139000000
C 0.804000000 9.683000000 19.694000000
C 0.409000000 11.806000000 20.774000000
C -0.564000000 9.378000000 19.787000000
H 1.475000000 8.948000000 19.222000000
C -1.477000000 10.291000000 20.379000000
H -0.941000000 8.429000000 19.390000000
C -0.963000000 11.515000000 20.881000000
N -2.837000000 9.914000000 20.481000000
H -1.642000000 12.240000000 21.342000000
H 0.777000000 12.763000000 21.175000000
C -3.907000000 10.814000000 20.459000000
N -5.144000000 10.194000000 20.658000000
O -3.755000000 12.035000000 20.289000000
C -6.406000000 10.835000000 20.759000000
C -7.616000000 10.057000000 20.627000000
C -6.523000000 12.228000000 20.998000000
C 8.362000000 7.471000000 30.954000000
H 9.262000000 5.502000000 30.664000000
C -8.868000000 10.716000000 20.729000000
N -7.521000000 8.661000000 20.400000000
C -8.953000000 12.102000000 20.945000000
H -9.773000000 10.112000000 20.621000000
C -7.776000000 12.858000000 21.083000000
H -9.941000000 12.581000000 21.013000000
H -7.824000000 13.943000000 21.263000000
H -5.602000000 12.808000000 21.096000000
C -8.511000000 7.815000000 19.923000000
N -8.007000000 6.516000000 19.706000000

O -9.691000000 8.134000000 19.700000000
C -8.737000000 5.355000000 19.494000000
C -7.992000000 4.173000000 19.179000000
C -10.160000000 5.258000000 19.594000000
C -8.630000000 2.953000000 18.982000000
H -6.901000000 4.255000000 19.106000000
C -10.037000000 2.879000000 19.095000000
H -8.060000000 2.048000000 18.749000000
C -10.796000000 4.031000000 19.401000000
N -10.710000000 1.601000000 18.894000000
C 8.540000000 8.749000000 31.572000000
N 7.214000000 7.298000000 30.187000000
H -11.886000000 3.947000000 19.484000000
H -10.744000000 6.155000000 19.818000000
C 10.459000000 11.834000000 29.249000000
C 11.071000000 12.998000000 29.767000000
C 9.109000000 11.604000000 29.495000000
H 11.052000000 11.130000000 28.656000000
C 10.325000000 13.924000000 30.530000000
N 12.485000000 13.243000000 29.510000000
C 8.971000000 13.695000000 30.783000000
H 10.823000000 14.820000000 30.921000000
C 8.330000000 12.528000000 30.263000000
H 8.393000000 14.402000000 31.384000000
N 6.986000000 12.224000000 30.425000000
H 8.608000000 10.713000000 29.098000000
C 6.034000000 12.935000000 31.186000000
N 4.744000000 12.537000000 30.869000000
O 6.340000000 13.795000000 32.028000000
P 5.220000000 9.908000000 28.227000000
C 3.538000000 12.957000000 31.485000000
C 3.471000000 14.126000000 32.285000000
C 2.338000000 12.174000000 31.292000000
H 7.761000000 9.508000000 31.443000000
C 2.271000000 14.531000000 32.895000000
H 4.389000000 14.703000000 32.424000000
C 1.102000000 13.775000000 32.701000000
H 2.257000000 15.441000000 33.514000000
C 1.138000000 12.618000000 31.905000000
H 0.152000000 14.083000000 33.163000000
H 0.236000000 12.020000000 31.754000000
N 2.388000000 10.999000000 30.500000000
C 1.441000000 9.973000000 30.472000000
N 1.756000000 8.968000000 29.553000000

O 0.419000000 9.944000000 31.181000000
C 1.573000000 6.763000000 28.568000000
C 0.947000000 7.855000000 29.231000000
C -0.446000000 7.760000000 29.493000000
C 0.846000000 5.622000000 28.205000000
H 2.644000000 6.824000000 28.348000000
C -0.539000000 5.509000000 28.476000000
H 1.381000000 4.801000000 27.705000000
C -1.161000000 6.606000000 29.114000000
C -1.326000000 4.256000000 28.069000000
H 9.818000000 10.021000000 32.783000000
H -2.239000000 6.562000000 29.332000000
H -0.957000000 8.580000000 30.005000000
C -1.515000000 4.139000000 26.557000000
H -2.314000000 4.272000000 28.570000000
H -0.790000000 3.357000000 28.438000000
C -0.563000000 3.479000000 25.748000000
C -2.607000000 4.762000000 25.904000000
C -0.634000000 3.499000000 24.343000000
H 0.276000000 2.947000000 26.222000000
C -1.677000000 4.211000000 23.698000000
H 0.129000000 2.994000000 23.743000000
C -2.694000000 4.794000000 24.504000000
N -1.753000000 4.439000000 22.305000000
H -3.531000000 5.302000000 24.009000000
H -3.397000000 5.253000000 26.492000000
C -0.675000000 4.515000000 21.436000000
N -0.935000000 5.289000000 20.315000000
O 0.427000000 3.956000000 21.659000000
C 6.759000000 6.091000000 29.625000000
N 5.652000000 6.264000000 28.811000000
O 7.305000000 4.993000000 29.826000000
C 1.353000000 5.488000000 19.335000000
C 2.176000000 5.790000000 18.237000000
C -0.060000000 5.554000000 19.228000000
H 1.802000000 5.198000000 20.288000000
C 1.608000000 6.182000000 17.011000000
H 3.268000000 5.728000000 18.351000000
C 0.211000000 6.268000000 16.889000000
H 2.247000000 6.433000000 16.152000000
C -0.638000000 5.948000000 17.974000000
H -0.244000000 6.575000000 15.941000000
N -2.054000000 6.016000000 17.845000000
C -2.746000000 5.538000000 16.739000000

N -4.138000000 5.627000000 16.889000000
O -2.192000000 5.068000000 15.733000000
P -4.476000000 6.472000000 20.623000000
C -5.096000000 5.148000000 16.001000000
C -4.820000000 4.285000000 14.894000000
C -6.453000000 5.531000000 16.242000000
C -5.860000000 3.823000000 14.085000000
H -3.790000000 3.975000000 14.701000000
C -7.191000000 4.211000000 14.350000000
H -5.655000000 3.148000000 13.246000000
C -7.489000000 5.068000000 15.434000000
N -8.273000000 3.722000000 13.498000000
H -8.528000000 5.359000000 15.625000000
H -6.670000000 6.198000000 17.085000000
C 11.529000000 7.811000000 27.113000000
C 10.882000000 6.624000000 27.523000000
C 9.473000000 6.516000000 27.473000000
N 11.670000000 5.506000000 28.027000000
C 10.776000000 8.907000000 26.686000000
H 12.624000000 7.865000000 27.149000000
C 9.348000000 8.838000000 26.678000000
H 11.265000000 9.833000000 26.371000000
C 8.720000000 7.607000000 27.051000000
N 8.510000000 9.893000000 26.364000000
H 7.625000000 7.542000000 27.030000000
H 8.990000000 5.587000000 27.795000000
C 8.855000000 11.214000000 26.031000000
N 7.719000000 12.021000000 26.037000000
O 10.008000000 11.590000000 25.777000000
H 5.177000000 3.722000000 29.607000000
C 7.629000000 13.401000000 25.773000000
C 8.745000000 14.189000000 25.389000000
C 6.345000000 14.035000000 25.894000000
C 8.595000000 15.555000000 25.094000000
H 9.721000000 13.702000000 25.318000000
C 7.332000000 16.169000000 25.186000000
H 9.477000000 16.139000000 24.790000000
C 6.222000000 15.411000000 25.594000000
H 7.210000000 17.238000000 24.957000000
H 5.234000000 15.877000000 25.683000000
N 5.223000000 13.279000000 26.321000000
C 3.921000000 13.400000000 25.814000000
N 3.048000000 12.539000000 26.463000000
O 3.614000000 14.172000000 24.891000000

C 0.852000000 13.339000000 25.555000000
C 1.647000000 12.490000000 26.368000000
C 0.987000000 11.536000000 27.191000000
C -0.552000000 13.269000000 25.635000000
H 1.343000000 14.059000000 24.894000000
H -1.149000000 13.954000000 25.012000000
C -1.214000000 12.374000000 26.504000000
H 2.716000000 8.934000000 29.128000000
H -2.581000000 6.424000000 18.656000000
H 7.478000000 9.752000000 26.560000000
H 3.472000000 11.953000000 27.229000000
N -6.276000000 3.405000000 22.369000000
H -5.607000000 4.200000000 22.273000000
N -5.011000000 2.710000000 20.584000000
H -4.576000000 3.654000000 20.775000000
H 6.752000000 8.207000000 29.893000000
H 5.224000000 7.220000000 28.706000000
H 4.622000000 7.593000000 26.853000000
H 5.260000000 9.108000000 25.487000000
H -3.014000000 8.883000000 20.392000000
H -5.117000000 9.174000000 20.882000000
H -6.662000000 8.175000000 20.768000000
H -6.953000000 6.408000000 19.600000000
O 11.995000000 9.482000000 33.756000000
O 12.747000000 7.453000000 33.372000000
O -11.956000000 1.551000000 19.013000000
O -10.017000000 0.598000000 18.609000000
O 13.007000000 14.288000000 29.962000000
O 13.128000000 12.399000000 28.846000000
H 6.681000000 11.305000000 29.980000000
H 4.654000000 11.987000000 29.974000000
H 3.131000000 10.963000000 29.765000000
H -2.689000000 4.804000000 21.970000000
H -1.801000000 5.888000000 20.350000000
H -4.530000000 5.952000000 17.822000000
O -7.987000000 2.968000000 12.541000000
O -9.444000000 4.075000000 13.757000000
O 12.918000000 5.611000000 28.047000000
O 11.071000000 4.483000000 28.429000000
H 6.838000000 11.504000000 26.256000000
H 5.339000000 12.595000000 27.083000000
N -5.188000000 7.651000000 24.271000000
C -4.597000000 8.749000000 24.919000000
C -5.900000000 6.609000000 24.849000000

H -5.168000000 7.667000000 23.218000000
N -6.475000000 5.774000000 23.880000000
C -7.347000000 4.687000000 24.153000000
H -6.274000000 6.024000000 22.902000000
C -7.262000000 3.485000000 23.372000000
C -6.065000000 2.378000000 21.454000000
C -4.631000000 2.077000000 19.413000000
N -3.467000000 0.539000000 15.593000000
O -4.045000000 -0.480000000 15.153000000
O -2.593000000 1.156000000 14.942000000
C -3.832000000 1.035000000 16.915000000
C -2.738000000 12.348000000 26.629000000
O -6.017000000 6.416000000 26.071000000
O -6.707000000 1.320000000 21.392000000
O 5.852000000 10.051000000 26.772000000
O 4.349000000 11.224000000 28.508000000
O 6.342000000 9.791000000 29.333000000
O 4.279000000 8.628000000 28.270000000
O -5.290000000 7.523000000 21.515000000
O -5.312000000 6.164000000 19.319000000
O -3.056000000 7.080000000 20.234000000
O -4.250000000 5.152000000 21.487000000
C -0.409000000 11.493000000 27.266000000
H -0.882000000 10.763000000 27.941000000
H 1.589000000 10.843000000 27.789000000
C -3.391000000 11.099000000 26.041000000
H -3.152000000 13.248000000 26.131000000
H -3.013000000 12.422000000 27.702000000
C -3.524000000 10.944000000 24.640000000
C -3.868000000 10.050000000 26.858000000
C -4.114000000 9.799000000 24.089000000
H -3.168000000 11.737000000 23.965000000
H -4.220000000 9.707000000 23.003000000
C -4.459000000 8.889000000 26.324000000
H -4.828000000 8.096000000 26.980000000
H -3.779000000 10.139000000 27.952000000
C -8.311000000 4.776000000 25.183000000
C -8.175000000 2.432000000 23.641000000
C -9.120000000 2.543000000 24.674000000
H -8.115000000 1.528000000 23.029000000
C -9.188000000 3.712000000 25.455000000
H -9.808000000 1.705000000 24.865000000
H -9.928000000 3.806000000 26.263000000
H -8.361000000 5.697000000 25.774000000

C -5.258000000 0.906000000 18.883000000

C -3.557000000 2.671000000 18.679000000

C -4.863000000 0.400000000 17.643000000

H -6.059000000 0.427000000 19.453000000

H -5.356000000 -0.481000000 17.217000000

C -3.165000000 2.164000000 17.444000000

H -2.368000000 2.642000000 16.863000000

H -3.059000000 3.559000000 19.088000000

2# Caged ACh$^+$

315

N -0.01700 8.00100 24.13900

C 0.77600 9.26300 23.84300

H 0.09100 10.12600 23.87400

H 1.23000 9.18800 22.84300

H 1.56100 9.38200 24.60800

C -1.07400 7.80100 23.06300

H -1.63200 6.87800 23.28200

H -0.59600 7.73800 22.07500

H -1.75700 8.66400 23.08400

C -0.71400 8.15800 25.48300

H -1.31400 7.25400 25.67700

H -1.36000 9.04800 25.44400

H 0.04900 8.27800 26.26800

C 0.90800 6.78400 24.24500

C 1.67800 6.42700 22.96800

H 1.60900 6.99400 25.07200

H 0.27700 5.93200 24.54600

H 2.45100 7.17100 22.70500

H 1.01600 6.29100 22.09300

O 2.30300 5.16400 23.28300

C 3.14000 4.64600 22.33400

C 3.70300 3.30800 22.76200

O 3.39600 5.22300 21.28900

H 3.85700 2.67200 21.87100

H 3.04500 2.80200 23.49100

H 4.68700 3.48900 23.23900

C 10.82700 8.73900 32.73200

C 10.77300 7.41600 32.24200

C 9.76600 9.64100 32.48600

N 11.98800 9.18300 33.49700

C 4.87700 2.79200 28.44900

C 4.43000 2.93100 27.12100

C 5.38600 3.91000 29.13200

H 4.81500 1.82100 28.96200

C 4.50400 4.17900 26.48000
H 4.00700 2.07000 26.58200
C 5.04800 5.30900 27.13800
H 4.13500 4.30500 25.45900
C 5.48600 5.17100 28.49900
N 5.13600 6.57800 26.51600
C 5.19500 6.83300 25.14300
N 5.20700 8.20200 24.87600
O 5.22800 5.95000 24.26800
C 4.81600 8.83000 23.67800
C 4.73100 8.18200 22.41900
C 4.43500 10.19800 23.75400
C 4.18800 8.86100 21.31200
H 5.05300 7.14200 22.32500
C 3.74000 10.19800 21.39700
H 4.08900 8.31200 20.36300
C 3.90900 10.86400 22.63600
C 3.02100 10.86800 20.22400
H 3.59800 11.91400 22.74900
C 9.66800 6.98300 31.50600
H 11.61300 6.73800 32.43700
H 4.52100 10.71500 24.71700
C 1.50400 10.65100 20.25000
H 3.42700 10.46900 19.27100
H 3.23000 11.95600 20.23500
C 0.91800 9.47300 19.72600
C 0.63400 11.59800 20.83800
C -0.46900 9.25900 19.77900
H 1.55000 8.70400 19.25700
C -1.33700 10.21500 20.37200
H -0.89500 8.34200 19.35500
C -0.75600 11.39600 20.90600
N -2.71800 9.91500 20.44200
H -1.39700 12.15700 21.36400
H 1.05300 12.52200 21.26600
C -3.73800 10.87000 20.49500
N -5.00700 10.30700 20.66500
O -3.51800 12.09000 20.40700
C -6.23400 11.00300 20.82600
C -7.48300 10.27800 20.76700
C -6.28000 12.40400 21.05100
C 8.58600 7.88000 31.23700
H 9.62900 5.96500 31.11200
C -8.69700 10.99400 20.92900

N -7.46700 8.87800 20.54500
C -8.71200 12.38400 21.13300
H -9.63200 10.42800 20.87800
C -7.49800 13.08900 21.19700
H -9.67400 12.90500 21.24900
H -7.48900 14.17600 21.36500
H -5.33100 12.94400 21.09300
C -8.52300 8.08000 20.13100
N -8.09000 6.76200 19.87300
O -9.70100 8.44800 19.98800
C -8.87900 5.63200 19.71800
C -8.21000 4.42400 19.33900
C -10.29100 5.59000 19.93900
C -8.91000 3.23000 19.19400
H -7.12700 4.46400 19.17300
C -10.30400 3.21000 19.42600
H -8.39700 2.30400 18.91400
C -10.98900 4.38900 19.79800
N -11.04000 1.96000 19.28300
C 8.66300 9.21200 31.75300
N 7.46400 7.56300 30.47900
H -12.07100 4.34700 19.97300
H -10.81900 6.50700 20.21300
C 10.27900 12.41200 29.23000
C 10.73500 13.68400 29.64600
C 8.96600 12.03700 29.49800
H 10.95900 11.74400 28.69300
C 9.87400 14.57100 30.32900
N 12.10800 14.08400 29.36200
C 8.55700 14.19600 30.60400
H 10.25200 15.55200 30.64100
C 8.07100 12.91800 30.18600
H 7.89000 14.87300 31.14500
N 6.77100 12.46800 30.37100
H 8.58300 11.06100 29.17600
C 5.73800 13.11000 31.08500
N 4.50300 12.55500 30.78300
O 5.94100 14.04400 31.87900
P 5.31600 9.85200 28.31600
C 3.25000 12.87000 31.36700
C 3.06200 14.04000 32.14700
C 2.12800 11.98000 31.16600
H 7.83300 9.90000 31.55400
C 1.82100 14.34700 32.73000

H 3.92300 14.69900 32.29100
C 0.72800 13.48800 32.52800
H 1.71600 15.26100 33.33300
C 0.88200 12.32700 31.75100
H -0.25500 13.71600 32.96800
H 0.04000 11.65000 31.59300
N 2.29200 10.80000 30.39500
C 1.39600 9.73000 30.30300
N 1.80900 8.74900 29.39700
O 0.33400 9.65200 30.94500
C 1.74600 6.61600 28.25000
C 1.06300 7.61900 28.99400
C -0.32000 7.41800 29.25500
C 1.08600 5.45600 27.81900
H 2.80700 6.76000 28.02100
C -0.28500 5.23600 28.09400
H 1.66500 4.70300 27.26300
C -0.96500 6.24900 28.80600
C -0.99500 3.95700 27.63000
H 9.82400 10.66400 32.87400
H -2.03600 6.12300 29.03200
H -0.87400 8.16900 29.82500
C -1.25300 3.91000 26.12300
H -1.95700 3.86500 28.17300
H -0.37800 3.08000 27.91700
C -0.32300 3.32600 25.23300
C -2.40300 4.51300 25.55600
C -0.47500 3.39600 23.83500
H 0.56500 2.81700 25.63800
C -1.58200 4.08500 23.27700
H 0.27600 2.96300 23.16800
C -2.57200 4.59500 24.16600
N -1.74600 4.36200 21.90400
H -3.45600 5.08700 23.74000
H -3.17700 4.94900 26.20700
C -0.72800 4.41800 20.94600
N -1.05200 5.28200 19.89800
O 0.33700 3.78300 21.03000
C 7.10100 6.28500 30.01700
N 5.99600 6.31100 29.18200
O 7.72000 5.25000 30.31400
C 1.16000 5.47600 18.77600
C 1.91900 5.81300 17.64200
C -0.25300 5.58700 18.76500

H 1.66000 5.12800 19.68300

C 1.28700 6.28900 16.47900

H 3.01500 5.71400 17.68200

C -0.11100 6.42500 16.45600

H 1.87700 6.56800 15.59300

C -0.89800 6.07000 17.57600

H -0.61600 6.80000 15.55900

N -2.31700 6.18700 17.54600

C -3.09600 5.79000 16.46800

N -4.47300 5.88300 16.72700

O -2.62600 5.37600 15.39500

P -4.52700 6.56200 20.51100

C -5.49900 5.42800 15.90500

C -5.31200 4.61800 14.74100

C -6.83400 5.77300 16.28500

C -6.41500 4.16100 14.01700

H -4.29900 4.34100 14.43900

C -7.72300 4.50400 14.42300

H -6.27800 3.52400 13.13500

C -7.93300 5.31600 15.56100

N -8.87000 4.01200 13.66500

H -8.95500 5.57500 15.86100

H -6.98400 6.40400 17.16800

C 11.74600 7.93900 27.47200

C 11.14100 6.74100 27.91200

C 9.74100 6.56500 27.82500

N 11.96300 5.68100 28.48400

C 10.95700 8.98000 26.97800

H 12.83500 8.04600 27.53700

C 9.53500 8.84300 26.92700

H 11.41400 9.91500 26.64000

C 8.95200 7.60100 27.33300

N 8.66100 9.84500 26.54100

H 7.86300 7.48100 27.28000

H 9.29200 5.62900 28.17600

C 8.96700 11.15400 26.13100

N 7.80800 11.92300 26.06500

O 10.11300 11.55100 25.87700

H 5.72400 3.81700 30.17000

C 7.69400 13.29300 25.75600

C 8.80500 14.09400 25.38100

C 6.39600 13.90400 25.82800

C 8.63800 15.44800 25.04800

H 9.79100 13.62500 25.34900

C 7.36100 16.04000 25.09300
H 9.51600 16.04100 24.75200
C 6.25600 15.27000 25.49100
H 7.22500 17.10100 24.83500
H 5.25800 15.72000 25.54400
N 5.27700 13.13800 26.24400
C 3.98100 13.24000 25.71900
N 3.10100 12.39000 26.37200
O 3.68200 13.99000 24.77500
C 0.91500 13.20200 25.45000
C 1.70000 12.35300 26.27200
C 1.02900 11.41700 27.10600
C -0.49000 13.15500 25.53500
H 1.41400 13.90700 24.77800
H -1.07900 13.84000 24.90500
C -1.16200 12.28200 26.41900
H 2.80300 8.75300 29.05500
H -2.77400 6.55400 18.41800
H 7.63100 9.67500 26.72100
H 3.51300 11.81500 27.15300
N -6.21800 3.35900 22.20500
H -5.56900 4.17000 22.10200
N -5.08800 2.79700 20.28800
H -4.64300 3.72800 20.50700
H 6.93900 8.41000 30.11400
H 5.49000 7.21900 29.01000
H 4.95200 7.39900 27.14200
H 5.39400 8.85600 25.68000
H -2.95500 8.90000 20.29900
H -5.02600 9.28000 20.85100
H -6.61100 8.34800 20.86000
H -7.05300 6.61700 19.67600
O 12.01500 10.36200 33.91400
O 12.91500 8.36900 33.70700
O -12.27000 1.95500 19.51600
O -10.41400 0.93400 18.93200
O 12.48800 15.22500 29.71400
O 12.86000 13.27200 28.77600
H 6.58000 11.49000 29.99400
H 4.49100 11.95900 29.91300
H 3.07400 10.80100 29.70200
H -2.68100 4.77700 21.65600
H -1.88600 5.90500 20.03800
H -4.78900 6.16800 17.70100

O -8.66200 3.29700 12.66000
O -10.01900 4.32200 14.05100
O 13.20400 5.83900 28.52300
O 11.39700 4.65500 28.92400
H 6.93800 11.39300 26.28900
H 5.38700 12.47500 27.02500
N -5.09500 7.54000 24.23100
C -4.51700 8.64800 24.87500
C -5.75300 6.47000 24.81800
H -5.10900 7.58600 23.17700
N -6.33900 5.63300 23.85600
C -7.17500 4.52000 24.13100
H -6.15500 5.88300 22.87700
C -7.13200 3.36500 23.27700
C -6.07100 2.39700 21.21100
C -4.79000 2.23500 19.05700
N -3.88700 0.93100 15.08200
O -4.52400 -0.03600 14.60800
O -3.03100 1.56600 14.42600
C -4.16200 1.34400 16.45500
C -2.68500 12.27600 26.54800
O -5.82200 6.25600 26.03900
O -6.70800 1.33700 21.12900
O 5.98000 9.92700 26.87000
O 4.33300 11.10600 28.47500
O 6.41000 9.91100 29.45400
O 4.47900 8.50500 28.44000
O -5.23600 7.61500 21.48500
O -5.45700 6.33100 19.25400
O -3.11500 7.13500 20.05000
O -4.30100 5.20300 21.31300
C -0.36800 11.39700 27.18900
H -0.84800 10.68500 27.87700
H 1.62300 10.72500 27.71300
C -3.34200 11.02300 25.97500
H -3.09200 13.17200 26.03800
H -2.96000 12.36600 27.62000
C -3.47600 10.85500 24.57500
C -3.80500 9.97500 26.80200
C -4.04900 9.69800 24.03600
H -3.13200 11.64700 23.89200
H -4.15800 9.59200 22.95200
C -4.38000 8.80100 26.27900
H -4.73500 8.00700 26.94200

H -3.71500 10.07500 27.89500
C -8.06900 4.53600 25.22700
C -8.01200 2.28500 23.54700
C -8.88800 2.32300 24.64400
H -7.98500 1.42000 22.87900
C -8.91400 3.44500 25.49300
H -9.55300 1.46700 24.83200
H -9.59900 3.48300 26.35300
H -8.08800 5.42000 25.87300
C -5.47300 1.11500 18.49100
C -3.74600 2.85400 18.30100
C -5.16400 0.68400 17.19900
H -6.25000 0.61400 19.07600
H -5.70300 -0.15700 16.74700
C -3.43700 2.42000 17.01500
H -2.66500 2.91900 16.42000
H -3.20300 3.70300 18.73600
